# Synthesis and properties of lysosome-specific photoactivatable probes for live-cell imaging†
†Electronic supplementary information (ESI) available. See DOI: 10.1039/c5sc01601k



**DOI:** 10.1039/c5sc01601k

**Published:** 2015-06-17

**Authors:** Mai N. Tran, Robert-André F. Rarig, David M. Chenoweth

**Affiliations:** a Department of Chemistry , University of Pennsylvania , 231 South 34th Street , Philadelphia , PA 19104-6323 , USA . Email: dcheno@sas.upenn.edu; b Department of Chemistry , Temple University , 130 Beury Hall, 1901 N. 13th Street , Philadelphia PA 19122 , USA

## Abstract

Fluorescent dyes have become increasingly important in cell biology since they enable high signal-to-noise and selectivity in visualizing subcellular organelles. Photoactivatable dyes allow for tracking and monitoring of a subset of cells or organelles. Here, we report the synthesis and application of a new class of large Stokes shift fluorescent dyes that are water-soluble, cell permeable, non-cytotoxic, and lysosome-specific. Additionally, we demonstrate temporally controlled sequential photoactivation of individual cells in close spatial proximity.

## Abstract 2

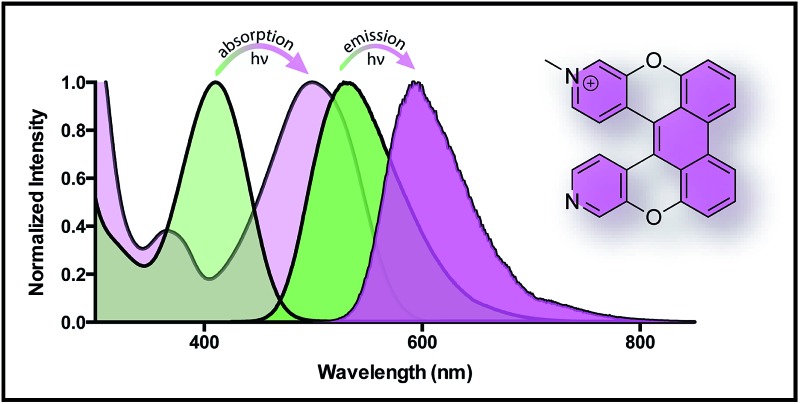
We describe the synthesis and application of a new class of large Stokes shift lysosome-specific photoactivatable probes for live-cell imaging.

## Introduction

Fluorescence microscopy is a powerful tool that is universally employed to study biological processes at the cellular level.^1–3^ Many fluorescent dyes targeting a multitude of organelles and subcellular targets have been developed.^4,5^ Photoactivatable dyes are an important but rare class of probes allowing for spatial and temporal control during imaging studies.^6–11^ Photoactivatable dyes can be grouped into two broad categories, the first switching from a dark state to a fluorescent state and the second converting from one fluorescent state to another fluorescent state.^12–14^ The later are often referred to as photoconvertible dyes. Each category has its own merit depending on the experimental conditions. Photoconvertible dyes have the added advantage of being able to track the pre-activated state, although few examples of useful dyes in this category currently exist.^15,16^ A combined Cy5-Cy3 probe was introduced by Johnsson *et al.* in 2010 as a photoconvertible protein label.^14^ In 2013, cell tracking experiments were performed using a commercial membrane stain DiR.^17^ Herein, we report a new photoconvertible lysosomal dye based on a diazaxanthilidene scaffold. The fluorescent probe is water-soluble, cell permeable, and noncytotoxic with a large Stokes shifts for both the pre- and post-activated forms.

In previous studies, we determined that the molecular structure of the natural product xylopypridine A was inconsistent with that of diazaxanthilidene **(*E*)-1**.^18^ During these studies we made several important observations about the photophysical and photochemical properties of **(*E*)-1**/**(*Z*)-1**.^18^ We also discovered that methylation of the pyridine ring led to water-soluble derivatives, facilitating biological experiments. In this report, we show that a simple switch in solvent produces two different derivatives, both of which can be used as lysosomal fluorescent probes for live cell imaging experiments. Importantly, we show that the monomethylated derivative can be photoactivated in cells, allowing for spatial and temporal control during the imaging process. Additionally, these new fluorescent probes are cell permeable and non-cytotoxic with good photostability and large Stokes shifts, facilitating applications in biological imaging experiments.

## Results and discussion

**Scheme 1:**
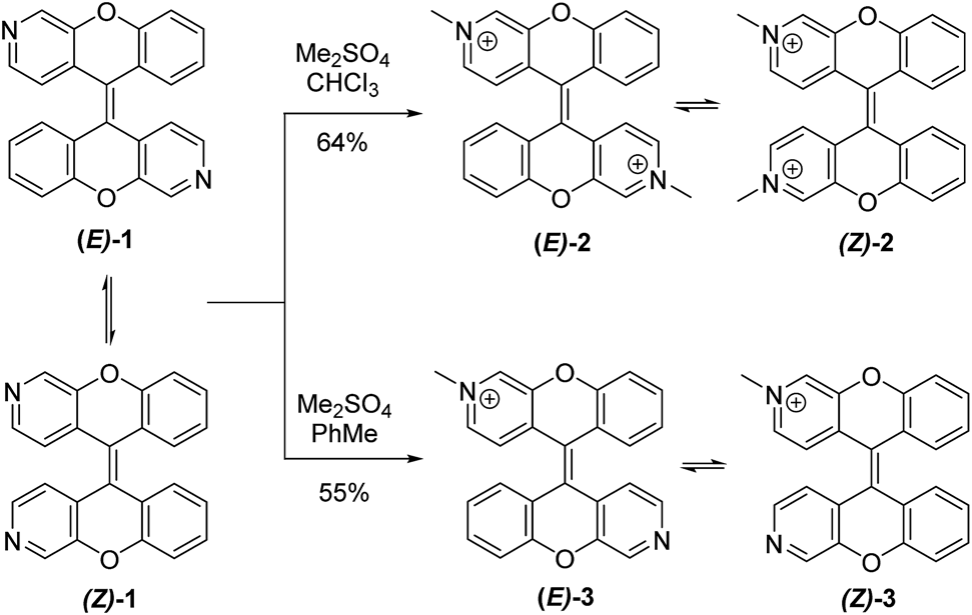
Synthesis of **(*E*)-2**/**(*Z*)-2** and **(*E*)-3**/**(*Z*)-3**.

We developed an efficient 5 step synthesis of **(*E*)-1**/**(*Z*)-1** resulting in a 41% overall yield.^18^ Treatment of **(*E*)-1**/**(*Z*)-1** with an excess of dimethyl sulfate in chloroform provided dimethylated derivatives **(*E*)-2**/**(*Z*)-2** in 64% yield, while one equivalent of dimethyl sulfate in toluene gave rise to the monomethylated derivatives **(*E*)-3**/**(*Z*)-3** in 55% yield (Scheme 1). Both methylated forms are isolated as a mixture of dynamic equilibrating *E* and *Z* isomers.

**Fig. 1:**
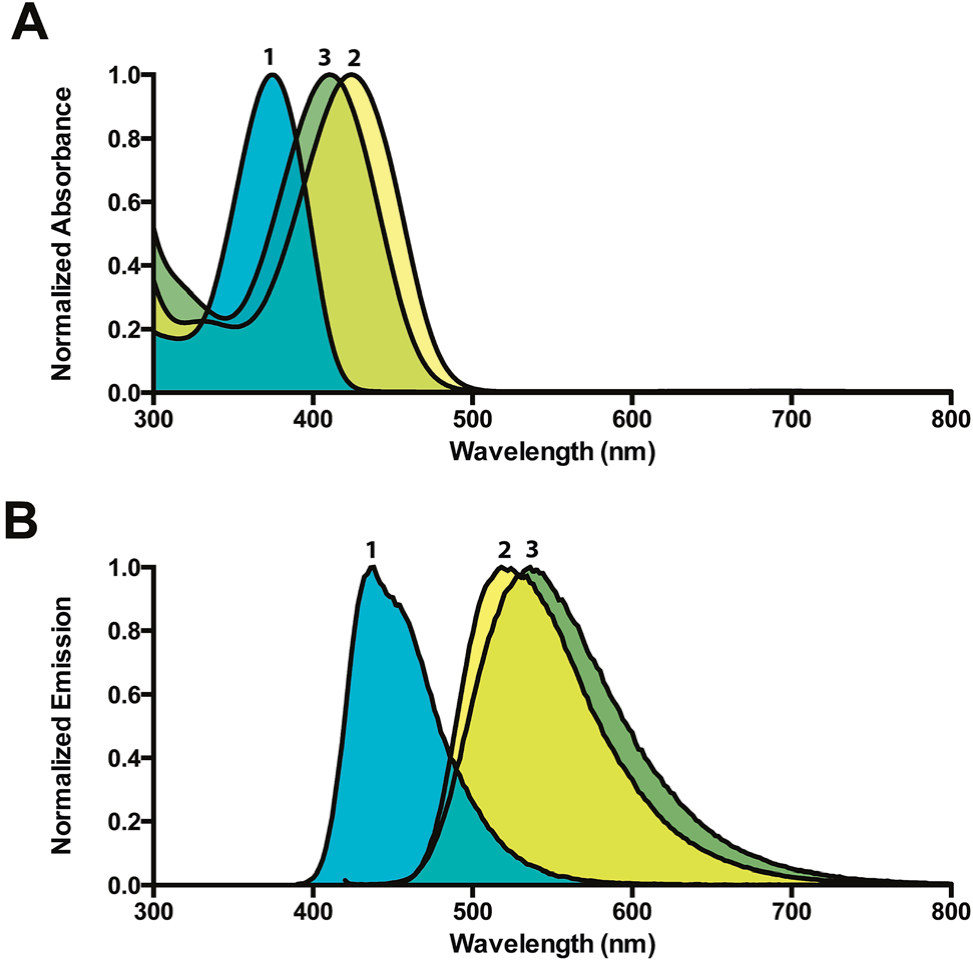
(A) Absorption spectra of **(*E*)-1**/**(*Z*)-1**, **(*E*)-2**/**(*Z*)-2**, and **(*E*)-3**/**(*Z*)-3** (*λ*_max_ = 378 nm, 424 nm, and 410 nm, respectively). (B) Emission spectra of **(*E*)-1**/**(*Z*)-1**, **(*E*)-2**/**(*Z*)-2**, and **(*E*)-3**/**(*Z*)-3** (*λ*_max_ = 435 nm, 518 nm, and 536 nm, respectively). Spectra of **(*E*)-1**/**(*Z*)-1** were recorded in chloroform, while spectra of **(*E*)-2**/**(*Z*)-2**, and **(*E*)-3**/**(*Z*)-3** were recorded in water.

Similar to **(*E*)-1**/**(*Z*)-1**, the methylated products were fluorescent with large bathochromic shifts observed in both absorption (46 nm and 32 nm) and emission (83 nm and 101 nm) spectra. Both **(*E*)-2**/**(*Z*)-2** and **(*E*)-3**/**(*Z*)-3** were soluble in water and exhibited large Stokes shifts of 94 nm and 126 nm, respectively (Fig. 1).

**Fig. 2:**
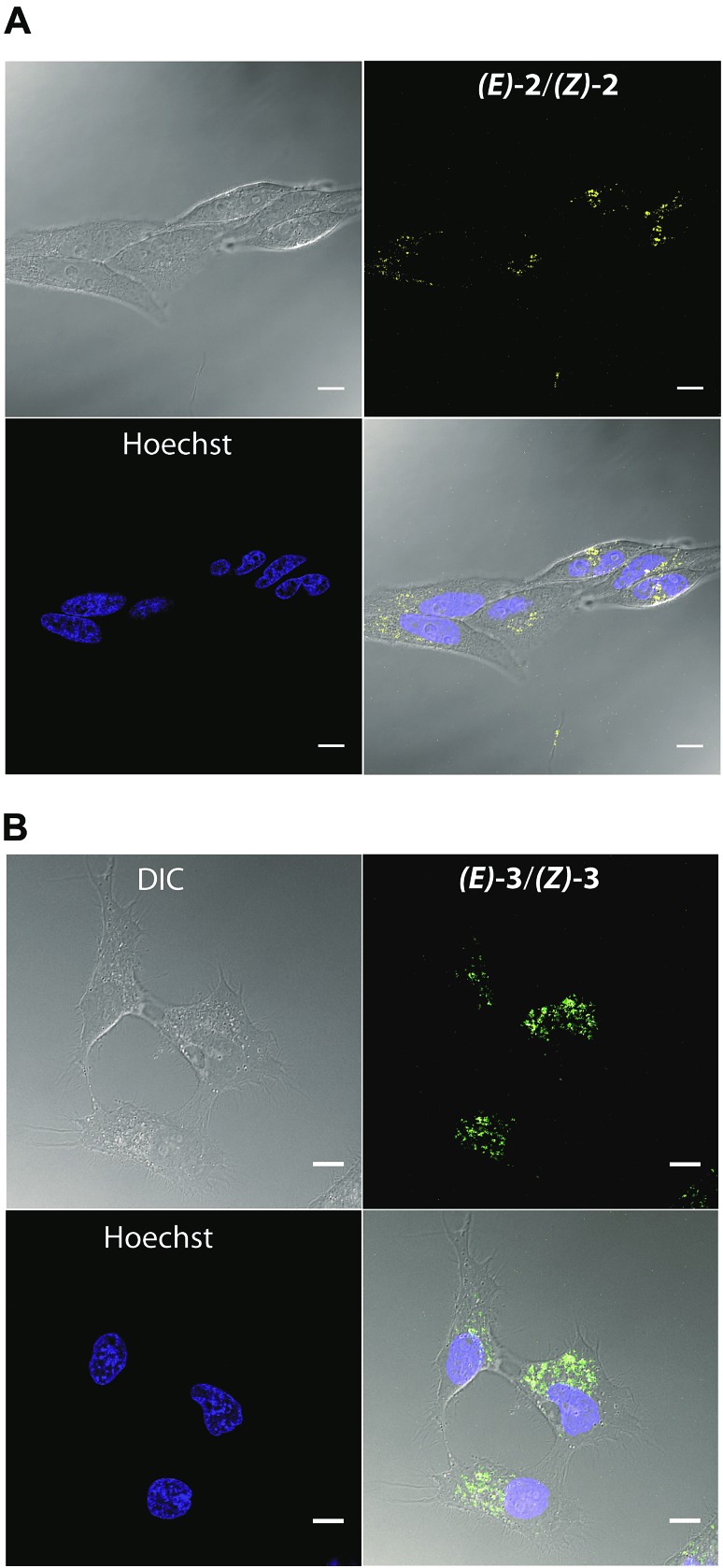
Differential interference contrast (DIC), fluorescence, and overlay images of HeLa cells stained with **(*E*)-2**/**(*Z*)-2** and **(*E*)-3**/**(*Z*)-3** and Hoechst 33342. The cells were first incubated with **(*E*)-2**/**(*Z*)-2** and **(*E*)-3**/**(*Z*)-3**, which were observed using 405/700 channel. Hoechst 33342 was then added and imaged after 10 minutes using 405/430 channel. Scale bar = 10 μm.

Live cell imaging studies were performed using **(*E*)-2**/**(*Z*)-2** and **(*E*)-3**/**(*Z*)-3**. Both compounds are water-soluble and can be dosed in water or buffer without the addition of organic solvents, which can be problematic for live cell imaging. After a 3 hour incubation in a humidified atmosphere with 5% CO_2_ at 37 °C, both dyes were found to be cell permeable and exhibited punctate staining patterns in HeLa cells, consistent with lysosomes (Fig. 2). Unlike **(*E*)-2**/**(*Z*)-2**, **(*E*)-3**/**(*Z*)-3** was found to be photoconvertible, a property that allowed for sequential labelling of individual cells (Fig. 5 and 6).

**Fig. 3:**
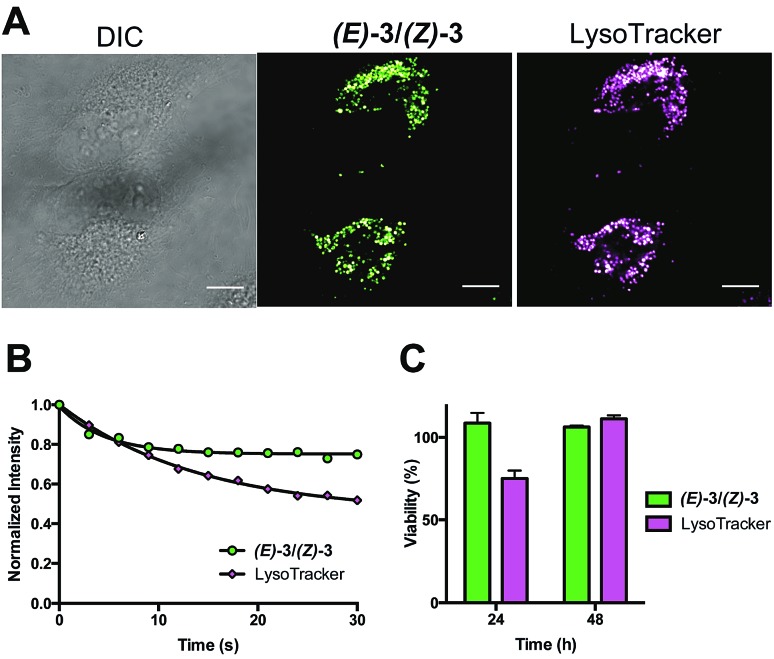
(A) DIC and fluorescence images of HeLa cells incubated with **(*E*)-3**/**(*Z*)-3** and LysoTracker. The 405/525 channel and 552/625 channel are used to observed **(*E*)-3**/**(*Z*)-3** and Lysotracker, respectively. Scale bar = 10 μm. (B) Normalized fluorescence intensity of **(*E*)-3**/**(*Z*)-3** and LysoTracker over 30 seconds of irradiation (100 laser pulses of 300 ms). The 444/495 channel and 594/632 channel are used to for **(*E*)-3**/**(*Z*)-3** and LysoTracker, respectively. Each set of data were fitted to a one phase exponential curve. The rate constant and half-life are 0.22 s^–1^ and 3.15 s for **(*E*)-3**/**(*Z*)-3** and 0.07 s^–1^ and 9.75 s for LysoTracker (C) cell viability experiments of HeLa cells incubated with **(*E*)-3**/**(*Z*)-3** and LysoTracker over 24 and 48 hours at 37 °C in a humidified atmosphere with 5% CO_2_.

To confirm lysosomal staining of **(*E*)-3**/**(*Z*)-3**, co-staining experiments with LysoTracker Red DND-99 were performed and the punctate localization patterns of **(*E*)-3**/**(*Z*)-3** were consistent with lysosome localization (Fig. 3A). Control experiments with **(*E*)-3**/**(*Z*)-3** alone and LysoTracker alone were also performed (Fig. S3†). Images of each sample were kept at the same brightness and contrast, with minimal bleed-through observed. Compared to LysoTracker Red DND-99,^19^ **(*E*)-3**/**(*Z*)-3** exhibited similarly low cytotoxicity (Fig. 3C) and higher photostability. Only a 20% decrease in intensity of **(*E*)-3**/**(*Z*)-3** was observed after 30 seconds of continuous irradiation, as opposed to 40% in LysoTracker (Fig. 3B). Imaging and bleaching studies were carried out using identical conditions, 405 nm excitation for **(*E*)-3**/**(*Z*)-3** and 594 nm excitation for LysoTracker.

**Fig. 4:**
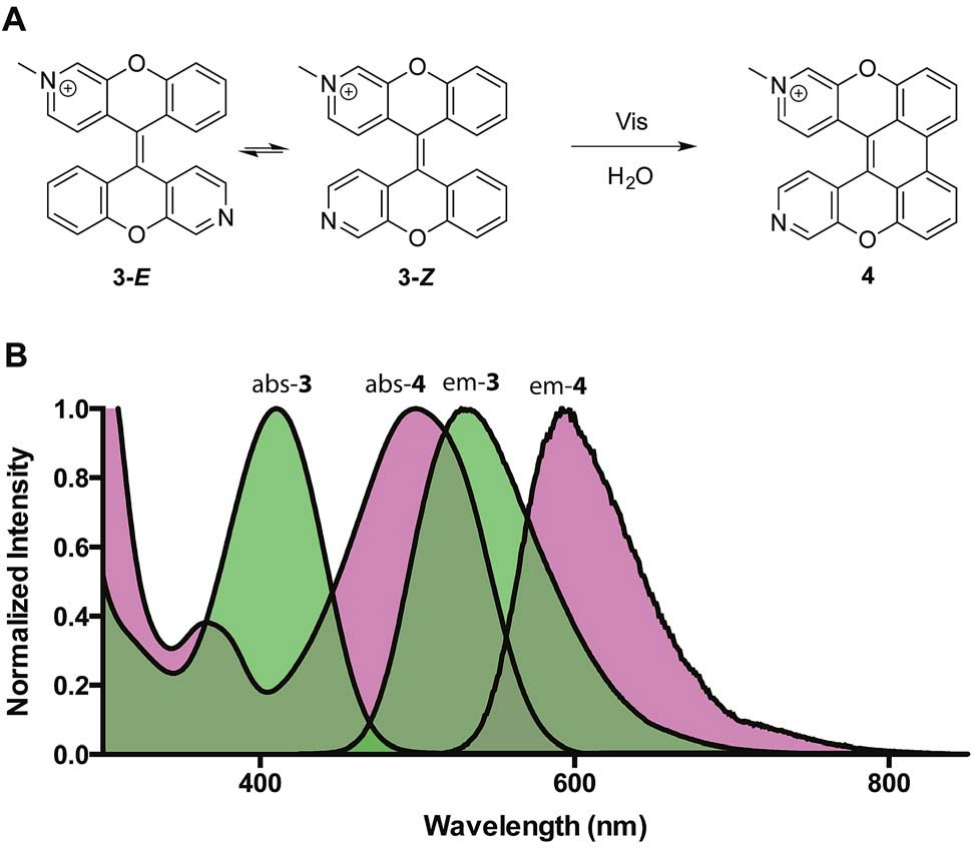
(A) Photoreaction of **(*E*)-3**/**(*Z*)-3**. (B) Absorption and emission spectra **(*E*)-3**/**(*Z*)-3** of and **4** (abs *λ*_max_ = 499 nm, em *λ*_max_ = 597 nm).

**Fig. 5:**
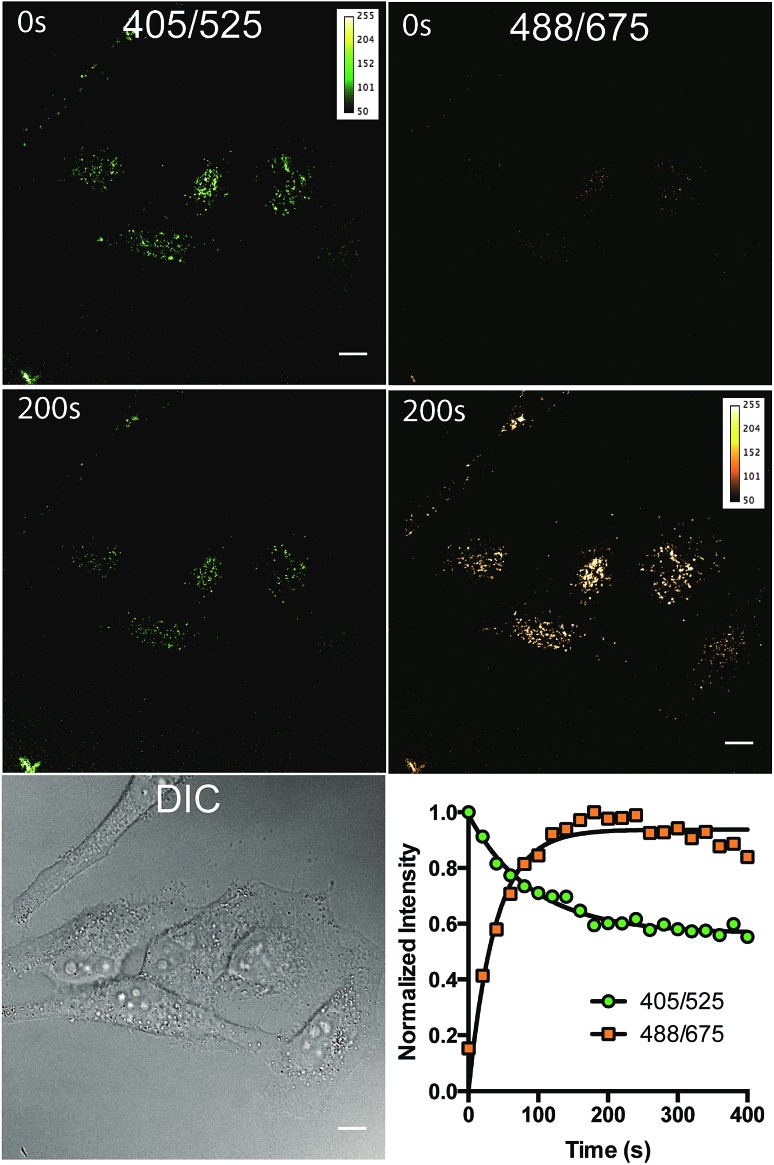
(A) DIC and fluorescence images of HeLa cells stained with **(*E*)-3**/**(*Z*)-3** and observed at 405/525 and 488/675 over 80 alternating 2.5 second pulses in a total of 200 seconds. (B) Normalized fluorescence intensity of the two channels 405/525 and 488/675 over 400 seconds of irradiation. Each data set were fitted to a one phase exponential curve. The rate constants and half-lives are 0.011 s^–1^ and 63.6 s for the 405/525 channel and 0.026 s^–1^ and 26.7 s for the 488/675 channel. The excitation and emission wavelength for imaging are 488 nm and 675 (±25) nm, respectively. Scale bar = 10 μm.

To study the photoreaction, a solution of **(*E*)-3**/**(*Z*)-3** in water was irradiated with visible light (26 W fluorescent light bulb) for 24 hours (Fig. 4A). Photoproduct **4** was isolated in 64% yield. The photoproduct **4** showed a bathochromic shift of 89 nm and a large Stokes shift (98 nm) (Fig. 4B). Photoactivation experiments were performed in live HeLa cells incubated with **(*E*)-3**/**(*Z*)-3**. The pre-activated **(*E*)-3**/**(*Z*)-3** can be observed using a 405 nm excitation wavelength and a 525 (±25) nm emission wavelength. The post-activated species can be excited at 488 nm and observed at 675 (±25) nm emission. Alternating 2.5 second pulses with 488 nm and 405 nm laser were used to investigate the photoconversion of **(*E*)-3**/**(*Z*)-3**. The photoproduct signal quickly increased, approaching its maximum at 180 seconds followed by a slow decrease to 80% after 400 seconds. The emission signal of the pre-activated state is reduced slowly followed by a plateau around 200 seconds at 60% of the original brightness (Fig. 5). The sharp increase in fluorescence signal of the post-activated form but slow decrease in fluorescence signal of the pre-activated form is a result of the relative brightness of the photoproduct compared to **(*E*)-3**/**(*Z*)-3**. Only 40% of **(*E*)-3**/**(*Z*)-3** was photoconverted but the increase in brightness of the photoproduct still allows for good signal detection over background. The remaining 60% of **(*E*)-3**/**(*Z*)-3** serves as a reference. This allows the ability to image both pre- and post-activated regions during imaging studies using two different fluorescent states as compared to traditional photoactivatable dyes with a pre-activated dark state and a post-activated fluorescent state. Similar experiment using 444 nm laser instead of 405 nm laser for **(*E*)-3**/**(*Z*)-3** excitation also showed photoactivation (Fig. S5†).

**Fig. 6:**
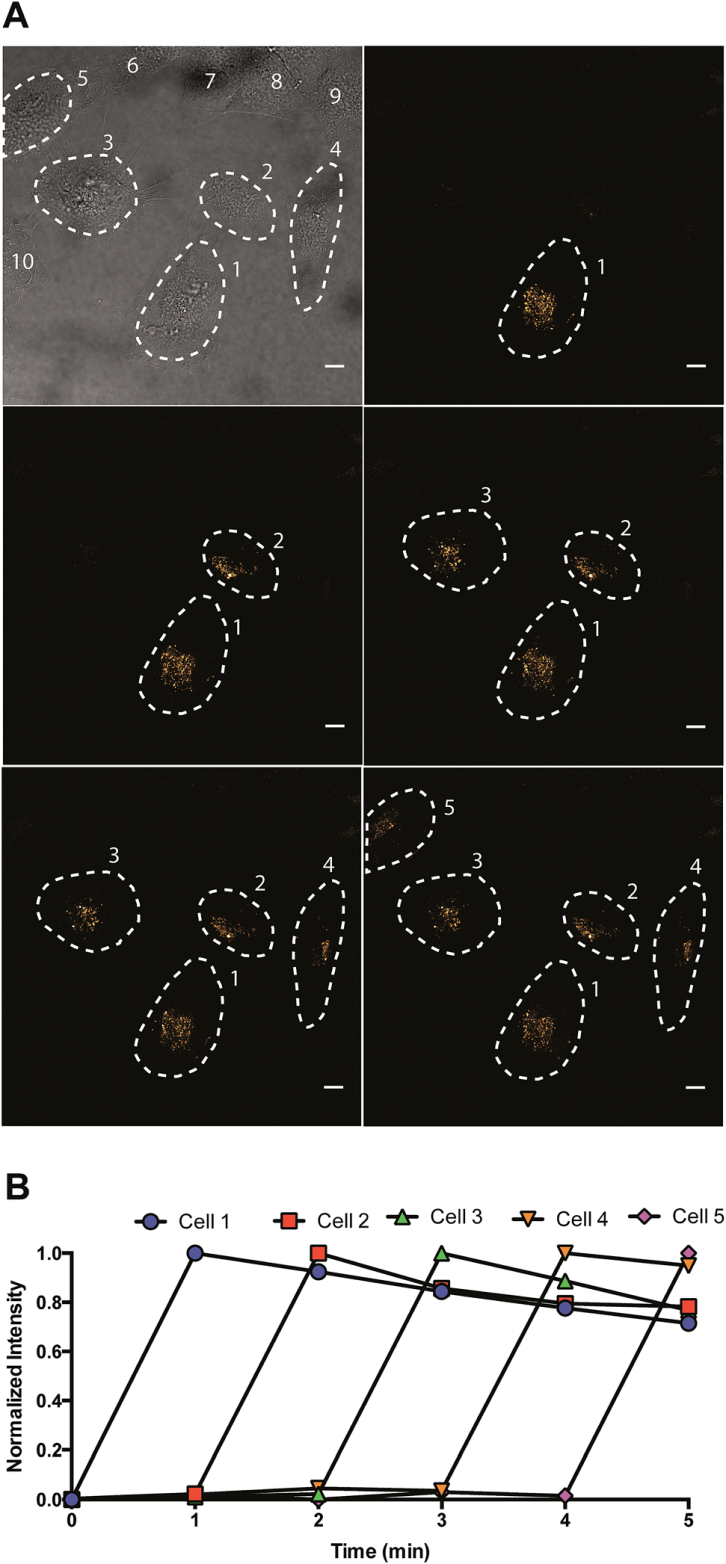
Sequential activation of five individual cells (cell 1, 2, 3, 4, and 5) in a field of ten HeLa cells. Dashed lines showed the cell periphery determined by DIC image. Each cell was activated by a 40 second irradiation using a 405 nm laser. The excitation and emission wavelength for imaging are 488 nm and 675 (±25) nm, respectively. Scale bar = 10 μm.

To investigate spatial selectivity, sequential activation was carried out with a dense population of HeLa cells. A 405 nm laser was used for photoconversion and the 488/675 channel was used to observe the post-activated state. Five individual cells (cell 1, 2, 3, 4, and 5) can be sequentially activated by 40 seconds of irradiation using a 405 nm laser (Fig. 6).

## Conclusions

In conclusion, a new class of water-soluble dixanthilidene fluorescent probe has been synthesized and used for lysosomal imaging in live cells. The monomethylated derivative can be photoconverted to a new fluorescent state, allowing precise spatiotemporal control during imaging experiments. These new fluorescent probes are cell permeable and photostable displaying large Stokes shifts and low cytotoxicity. More studies are under way in order to expand this new class of fluorophores, developing more organelle specific probes for live cell imaging and exploring further biological applications.
