# Supplementary material for: Synthesis and properties of lysosome-specific photoactivatable probes for live-cell imaging
Source: Chem Sci. 2015 Jun 17;6(8):4508–12. doi: 10.1039/c5sc01601k (PMC5299591; doi:10.1039/c5sc01601k)
Supplement: Supplementary file 1 [file SC-006-C5SC01601K-s001.pdf]

Electronic Supplementary Material (ESI) for Chemical Science.
This journal is © The Royal Society of Chemistry 2015

Synthesis and Properties of Lysosome-Specific Photoactivatable Probes for Live-Cell Imaging

Mai N. Tran, Robert-André F. Rarig and David M. Chenoweth*

Department of Chemistry, University of Pennsylvania, 231 34th Street, Philadelphia, Pennsylvania 19104-6323, United States

Table of content

General information....S2

Experimental Procedures....S3

References....S4

Absorption and emission spectra of ***(E)*-1/*(Z)*-1**, ***(E)*-2/*(Z)*-2**, ***(E)*-3/*(Z)*-3**, and **4** in different solvents....S5

Cell imaging....S7

Cytotoxicity studies....S7

Costaining experiments of ***(E)*-3/*(Z)*-3** with LysoTracker....S8

Photoactivation of ***(E)*-3/*(Z)*-3** using 405 nm laser....S9

Photoactivation of ***(E)*-3/*(Z)*-3** using 444 nm laser....S10

Sequential activation experiments of ***(E)*-3/*(Z)*-3**....S11

HPLC chromatogram of ***(E)*-2/*(Z)*-2**....S18

HPLC chromatogram of **4**....S19

HPLC chromatogram of ***(E)*-2/*(Z)*-2** after 2 months....S20

HPLC chromatogram of ***(E)*-3/*(Z)*-3** after 2 months....S21

Mass spectra....S22

NMR spectra....S24

General information

All reagents were purchased either from Sigma Aldrich or Acros Organics and used without any further purification. Anhydrous solvents were purchased from Fisher Scientific and passed through an alumina column of a solvent purification system. Column chromatography was performed using 230-400 mesh silica gel from Silicycle. Reversed phase column chromatography was carried out using a CombiFlash Rf System with a RediSep Rf Gold C18 column from Teledyne ISCO. High performance liquid chromatography (HPLC) was performed using a Phenomenex column (Luna 5u C18(2) 100A; 250 x 4.60 mm, 5 micron) on a Jasco HPLC system. ^{1}H-NMR and ^{13}C-NMR data were collected on a Bruker DMX 500 (500 MHz). High resolution mass spectrometry was performed by Dr. Rakesh Kohli at the Mass Spectrometry Facility of the Department of Chemistry, University of Pennsylvania using a Waters LCT Premier XE Mass Spectrometer (model KE 332). UV-Vis absorption spectra were recorded in a semi-micro 1-cm-pathlength quartz cuvette purchased from Starna Cells on a Jasco V-650 spectrophotometer. Fluorescence emission spectra were obtained using a micro square 1-cm-pathlength quartz fluorimeter cuvette from Starna Cells on a Horiba Jobin-Yvon FluoroLog.

Experimental Procedures

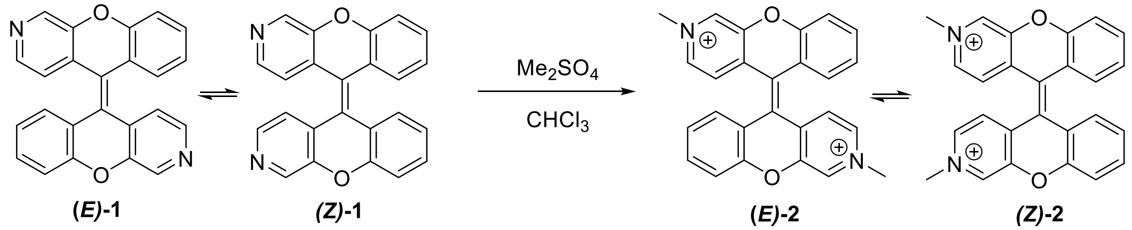

A mixture of ***(E)*-1/*(Z)*-1** (264 mg, 0.73 mmol) was dissolved in 15 mL chloroform. Dimethylsulfate (0.1 mL, 1.05 mmol) was added. The reaction mixture was heated under reflux for 24 hours. The reaction was allowed to cool down to room temperature before 50 mL water was added. The mixture was transferred to a separatory funnel to remove the organic layer. The aqueous layer was washed twice with 15 mL chloroform. Water was removed under vacuo and the residue was purified by reversed phase column chromatography using water containing 0.1% (v/v) trifluoroacetic acid and acetonitrile as eluents. ***(E)*-1/*(Z)*-1** was obtained as orange solid (279 mg, 0.45 mmol, 62%). ^{1}H NMR (500 MHz, $CDCl_3$): δ (ppm) 9.06 (0.6, s), 9.01 (2.0, s), 8.40 (0.6, d, J = 6.0 Hz), 8.32 (2.0, d, J = 6.5 Hz), 7.83 (2.6, overlapping doublet), 7.56 (4.0, m), 7.50 (1.2, m), 7.28 (0.6, d, J = 8.0 Hz), 7.23 (2.0, d, J = 7.5 Hz), 7.16 (2.0, m), 7.11 (0.6, m), 4.42 (1.8, s), 4.40 (6.0, s). ^{13}C NMR (126.9 MHz, $CDCl_3$): δ (ppm) 163.0, 162.7, 144.3, 153.3, 139.5, 139.4, 139.1, 136.9, 136.5, 131.8, 131.4, 127.8, 127.5, 125.3, 125.2, 125.1, 124.8, 122.8, 120.2, 119.6, 118.1, 117.6, 117.5, 115.2, 48.2, 48.1. HRMS (m/z): $[M]^{2+}$ calcd for $C_{26}H_{20}N_2O_2^{2+}$, 392.1516; found, 392.1536. Extinction coefficient ε = 11051 $M^{-1}cm^{-1}$ (solvent: water). Quantum yield ϕ = 0.176 (solvent: water, standard: 9,10-diphenylanthracene in cyclohexane).

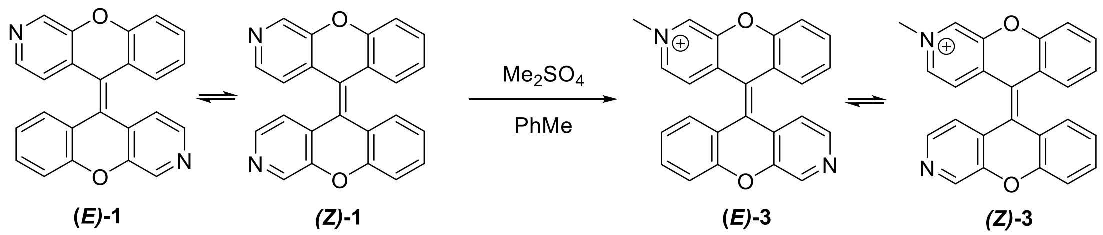

A mixture of ***(E)*-1/*(Z)*-1** (41 mg, 0.11 mmol) was suspended in 20 mL toluene. A 1% solution of dimethylsulfate (0.53 mL, 0.06 mmol) was added. The reaction mixture was heated under reflux for 12 hours. Another batch of dimethylsulfate (1% solution in toluene, 0.53 mL) was added. The reaction mixture was heated under reflux for an additional 24 hours before cooled down to room temperature. The precipitate was filtered and washed with toluene, dissolved in water and purified by reversed phase column chromatography using 0.1% TFA/water and acetonitrile as eluents. The product is a yellow solid (30 mg, 55%). Characterization data can be found in our previous study.[1] Extinction coefficient ε = 9867 $M^{-1}cm^{-1}$ (solvent: water). Quantum yield ϕ = 0.051 (solvent: water, standard: 9,10-diphenylanthracene in cyclohexane).

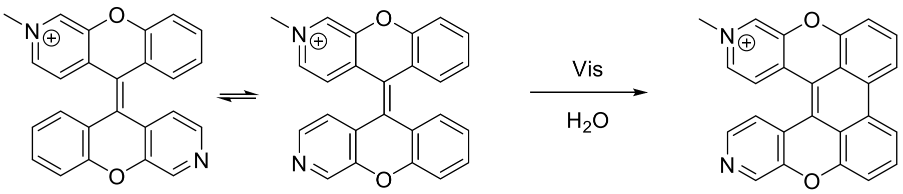

(*E*)-3 (*Z*)-3 4

A mixture of ***(E)*-3/*(Z)*-3** [1](17.2 mg, 0.035 mmol) was dissolved in 20 mL water. The reaction mixture was stirred and irradiated with a 26W fluorescent light bulb for 3 days. Water was removed under vacuo and the residue was purified by reversed phase column chromatography using 0.1% TFA/water and acetonitrile as eluents. Photoproduct **4** was obtained as red solid (10.9 mg, 0.023 mmol, 64%). ^{1}H NMR (500 MHz, $CDCl_3$): δ (ppm) 8.57 (1.0, s), 8.27 (1.0, s), 8.10 (1.0, d, J = 6.5 Hz), 7.93 (1.0, d, J = 5.5 Hz), 7.68 (1.0, d, J = 6.5 Hz), 7.51 (1.0, d, J = 8.0 Hz), 7.45 (1.0, d, J = 8.0 Hz), 7.38 (1.0, t, J = 8.0 Hz), 7.33 (1.0, t, J = 8.0 Hz), 7.25 (1.0, d, J = 5.5 Hz), 7.01 (1.0, d, J = 8.0 Hz), 6.87 (1.0, d, J = 8.0 Hz), 4.29 (3.0, s). ^{13}C NMR (126.9 MHz, $CDCl_3$): δ (ppm) 162.9, 162.6, 150.2, 148.7, 147.5, 147.2, 138.8, 135.8, 130.5, 130.0, 128.3, 122.8, 120.7, 118.2, 117.9, 117.7, 117.5, 115.2, 114.3, 113.6, 113.4, 47.8. HRMS (m/z): $[M]^+$ calcd for $C_{25}H_{15}N_2O_2^+$, 375.1134; found, 375.1139. Extinction coefficient ε = 10437 $M^{-1}cm^{-1}$ (solvent: water). Quantum yield ϕ = 0.101 (solvent: water, standard: fluorescein in 0.1N NaOH).

<u>References</u>

1. R. Rarig, M. Tran, D. Chenoweth, *J. Am. Chem. Soc.* 2013, **135**, 9213.

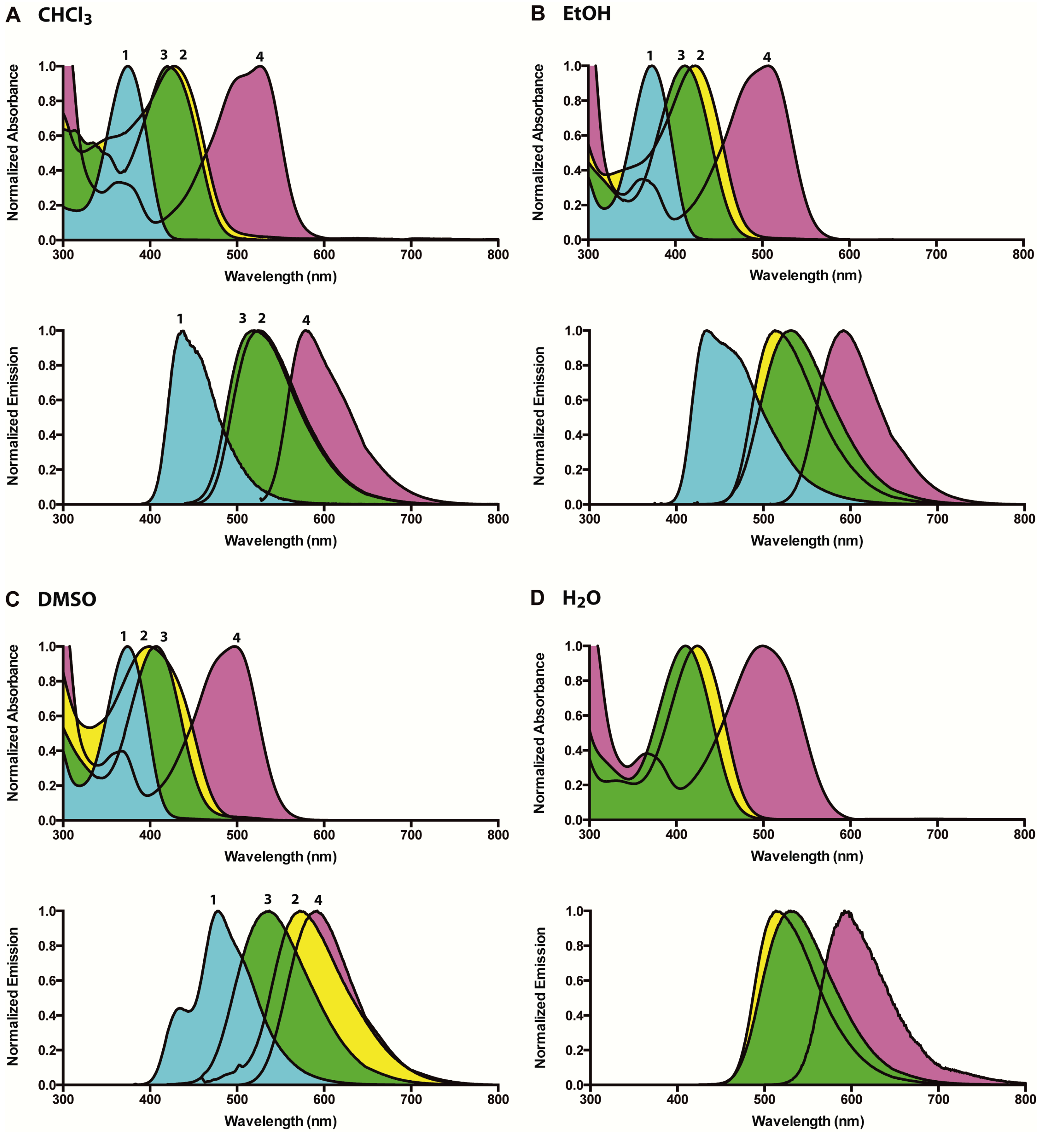

Figure S1: Absorption and emission spectra of ***(E)*-1/*(Z)*-1 (blue)**, ***(E)*-2/*(Z)*-2 (yellow)**, ***(E)*-3/*(Z)*-3 (green)**, and **4 (purple)** in (A) chloroform, (B) ethanol, (C) dimethylsulfoxide, and (D) water.

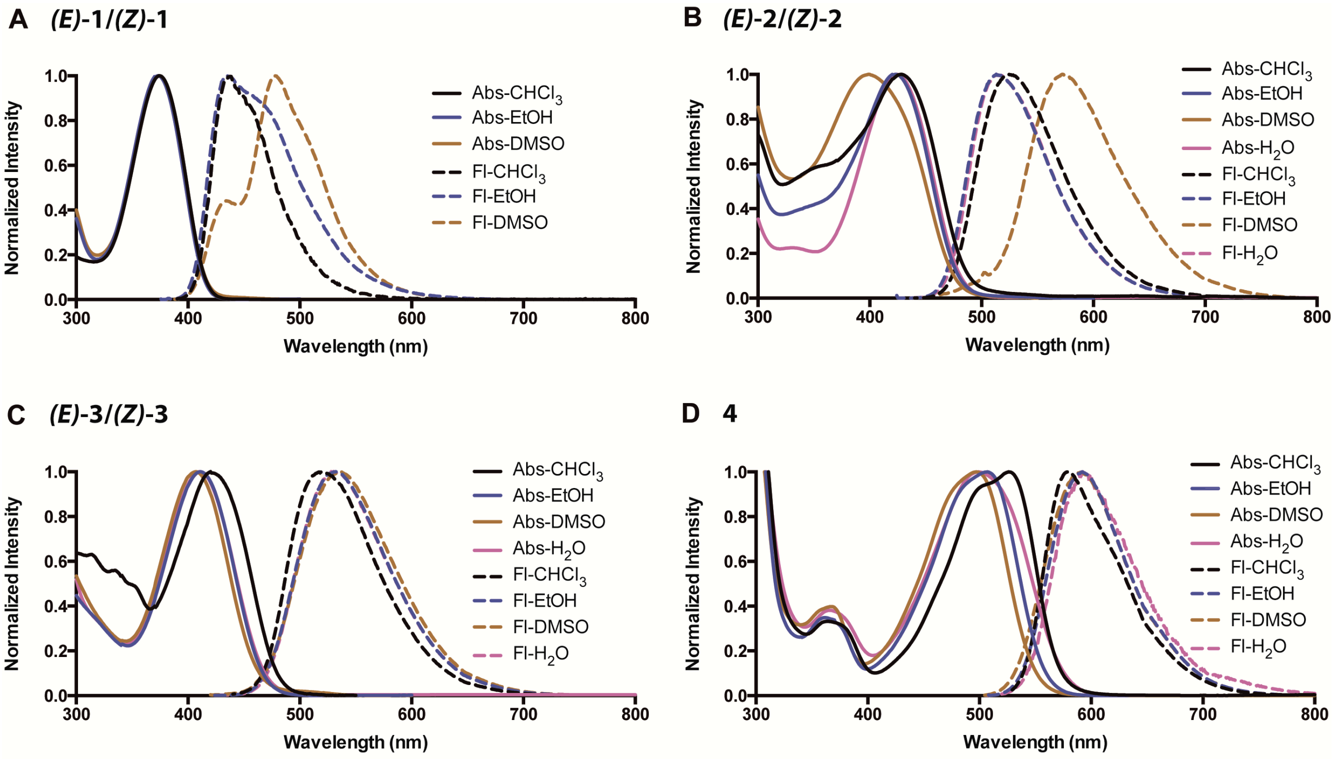

E

	$CHCl_3$	EtOH	DMSO	H_2O
***(E)*-1/*(Z)*-1**	378/435	373/435	374/478	
***(E)*-2/*(Z)*-2**	428/524	423/513	399/537	424/518
***(E)*-3/*(Z)*-3**	420/520	411/532	407/573	410/536
**4**	526/579	507/592	497/592	499/597

Figure S2: Absorption and emission spectra of (A) ***(E)*-1/*(Z)*-1**, (B) ***(E)*-2/*(Z)*-2**, (C) ***(E)*-3/*(Z)*-3**, and (D) **4** in chloroform, ethanol, dimethylsulfoxide, and water. (E) Summary of λ_{max} absorption/λ_{max} emission of ***(E)*-1/*(Z)*-1**, ***(E)*-2/*(Z)*-2**, ***(E)*-3/*(Z)*-3**, and **4** in different solvents. Absorption and emission spectra of ***(E)*-1/*(Z)*-1** in water could not be recorded because of low solubility.

Cell imaging

HeLa cells were cultured in a 60 mm culture dish using Dulbecco's Modified Eagle Medium (DMEM) media with phenol red, 10 % Fetal Bovine Serum and 1% penicillin-streptomycin purchased from Life Technologies at 37 ^{o}C under a humidified atmosphere with 5% CO_2. At least one day before imaging, the cells were detached using 0.25% trypsin-EDTA and transferred to a MatTek 35 mm glass bottom poly-D-lysine coated dish and incubated in 2 mL of the above mentioned media at 37 ^{o}C under a humidified atmosphere with 5% CO_2. Before imaging, cells were incubated with ***(E)*-2/*(Z)*-2** (5 mM stock solution in water, 5 µM final concentration, 3 to 12 hours), ***(E)*-3/*(Z)*-3** (5 mM stock solution in water, 5 µM final concentration, 3 to 12 hours), or LysoTracker Red DND-99[1] (1 mM stock solution in DMSO, 1 µM final concentration, 1 hour) at 37 ^{o}C. Excess dyes were removed by washing twice with non-phenol red DMEM before imaging. Cells were kept in 1 mL non-phenol red DMEM during imaging. Images were obtained from either Leica DM4000 spinning disk confocal microscope equipped with a 100x/1.4 NA oil immersion objective (for cell imaging experiments in Figure S12), or a Leica TCS SP8 confocal microscope equipped with a 63x/1.4 NA oil immersion objective lens (for other cell imaging experiments). Excitation and emission wavelengths for each sample were included in figures or figure captions.

Cytotoxicity studies

Cytotoxicity studies were performed in a 96-well plate. HeLa cells were detached from cell culture dish using 0.25% trypsin-EDTA and transfer to a falcon tube. Cell concentration was determined using a hemocytometer with Trypan Blue stain to exclude dead cells. Cell suspension was then diluted to 100,000 cells/mL and loaded to wells (50 µL/well). After 24 hours, ***(E)*-3/*(Z)*-3** in water (5 µM final concentration), LysoTracker Red DND-99 in DMSO (1 µM final concentration), and DMSO (vehicle control for LysoTracker, 1 µM final concentration) were added and the plate was incubated at 37 ^{o}C under a humidified atmosphere with 5% CO_2. The final volume was 100 µL/well. After 22 hours and 46 hours, Alamar Blue (10 µL) was added to each well and the plate was incubated at the above-mentioned condition for 2 hours. Cell viability was checked using fluorescence intensity (560 nm excitation and 590 nm emission) on a Tecan M1000 plate reader.

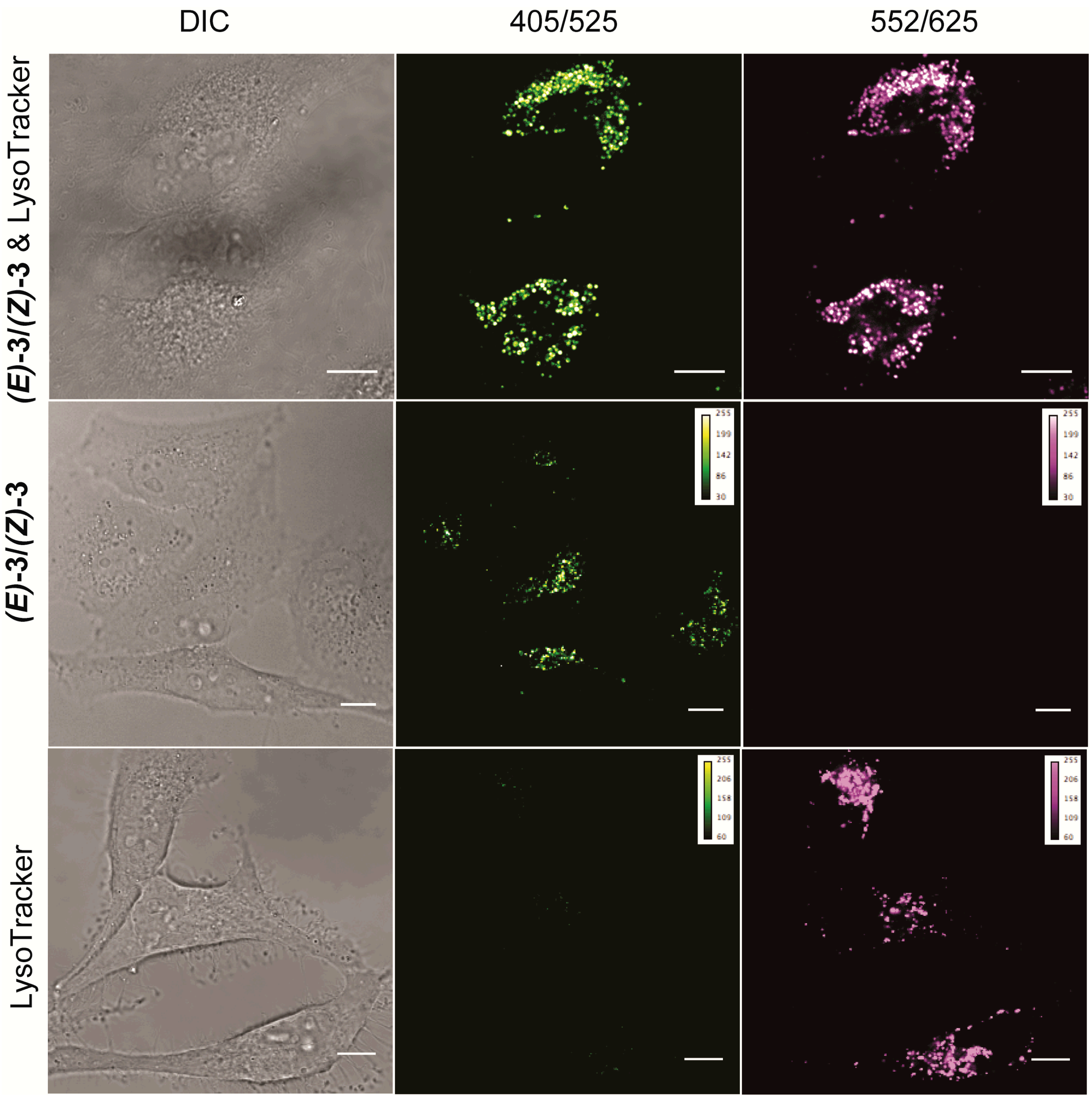

Figure S3: Costaining experiments of ***(E)*-3**/***(Z)*-3** and LysoTracker. DIC and fluorescence images of HeLa cells incubated with ***(E)*-3**/***(Z)*-3** and LysoTracker (top panel), ***(E)*-3**/***(Z)*-3** only (middle panel), and LysoTracker only (bottom panel). The 405/525 channel and 552/625 channel are used to observed ***(E)*-3**/***(Z)*-3** and Lysotracker, respectively. All images of each sample were kept at the same brightness and contrast. Almost no bleed-through was observed. Scale bar = 10 μm.

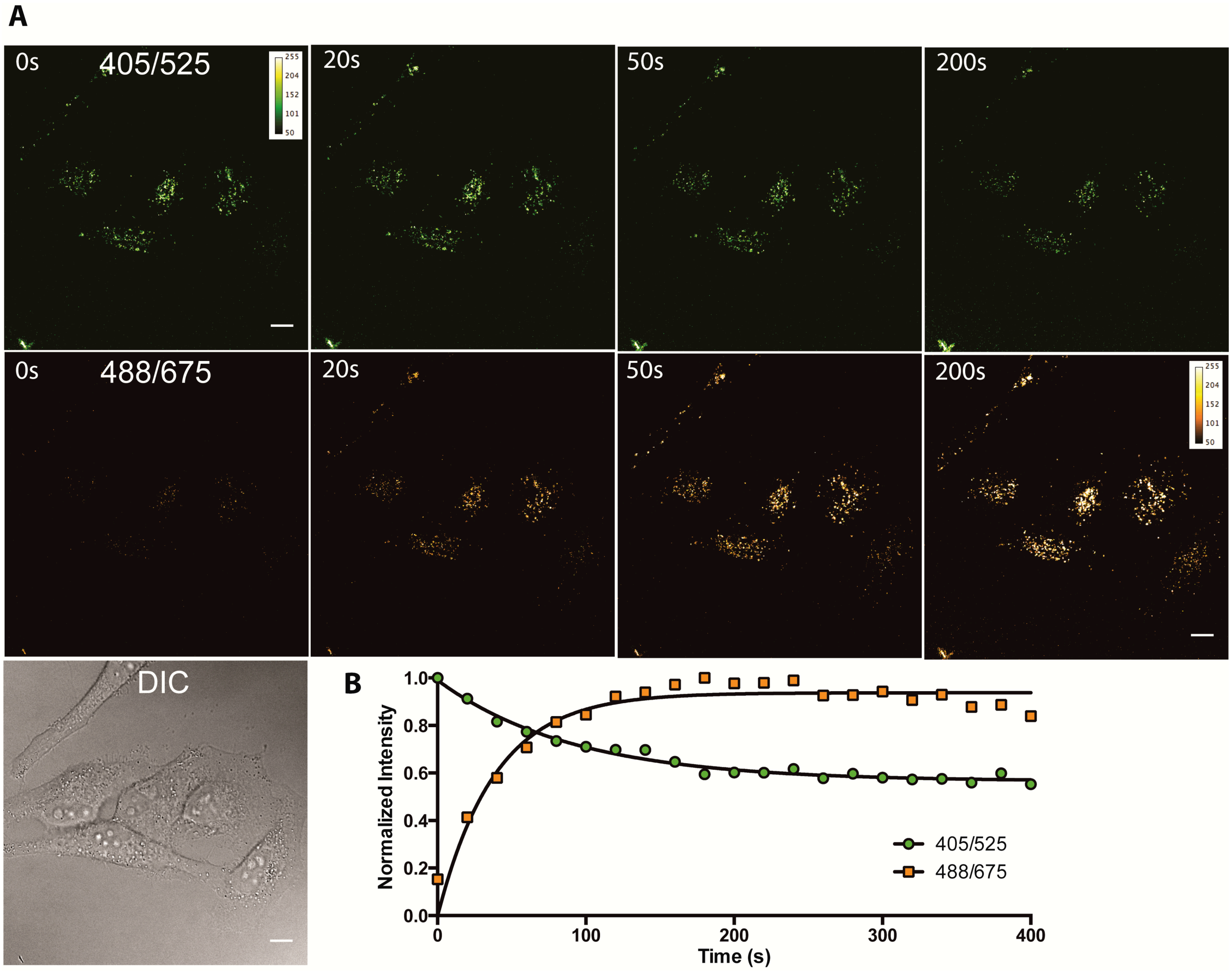

Figure S4: (A) DIC and fluorescence images of HeLa cells stained with ***(E)*-3**/***(Z)*-3** and observed at 405/525 and 488/675 over 80 alternating 2.5-second pulses in a total of 200 seconds. Scale bar = 10 µm. (B) Normalized fluorescence intensity of the two channels 405/525 and 488/675 over 400 seconds of irradiation.

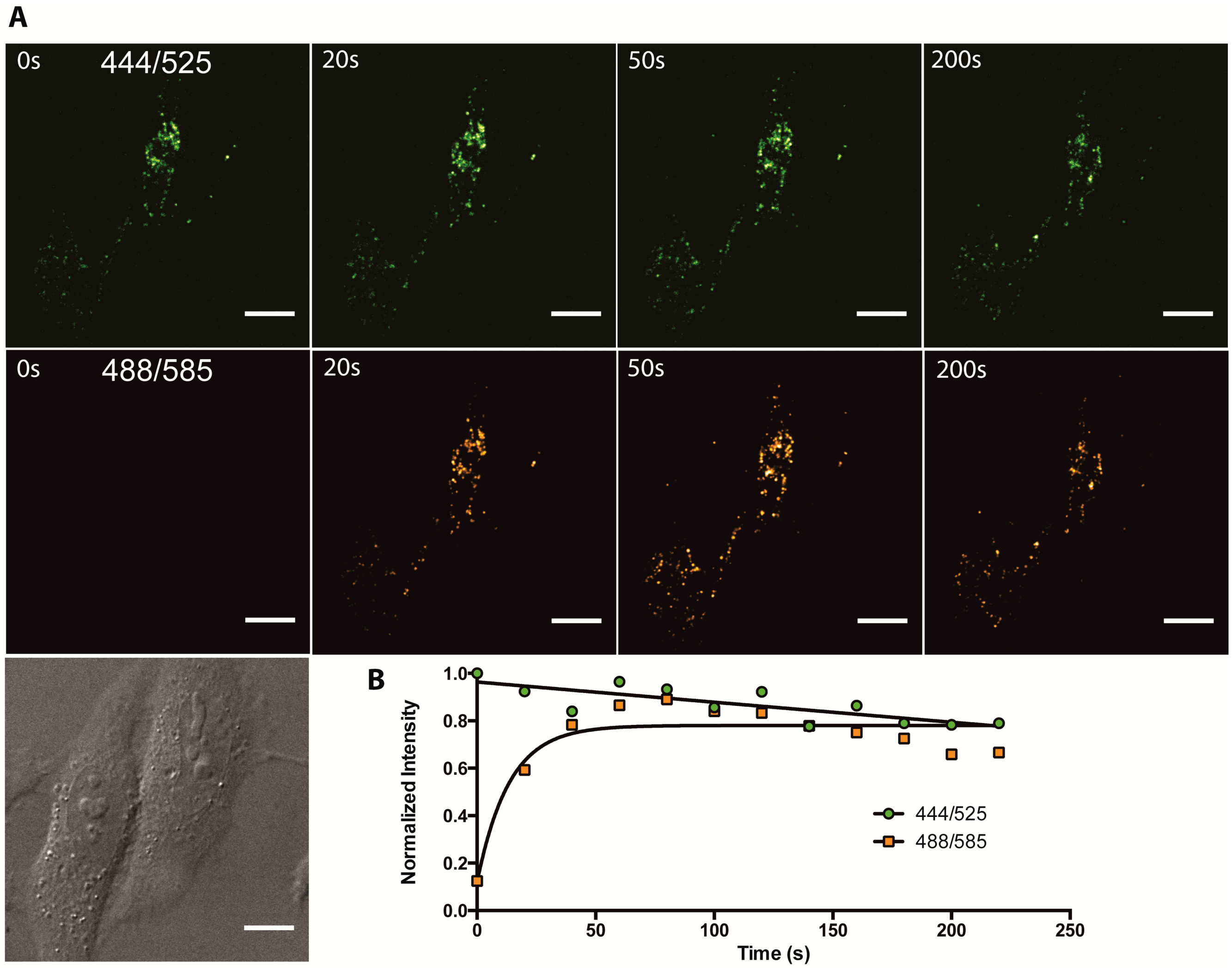

Figure S5: (A) DIC and fluorescence images of HeLa cells stained with ***(E)*-3**/***(Z)*-3** and observed at 444/525 and 488/585 over 1200 alternating 200-milisecond pulses in a total of 240 seconds. Scale bar = 10 µm. (B) Normalized fluorescence intensity of the two channels 444/525 and 488/585 over 240 seconds of irradiation.

__Sequential activation experiments of *(E)*-3/*(Z)*-3 (enlarged from Figure 6A)__

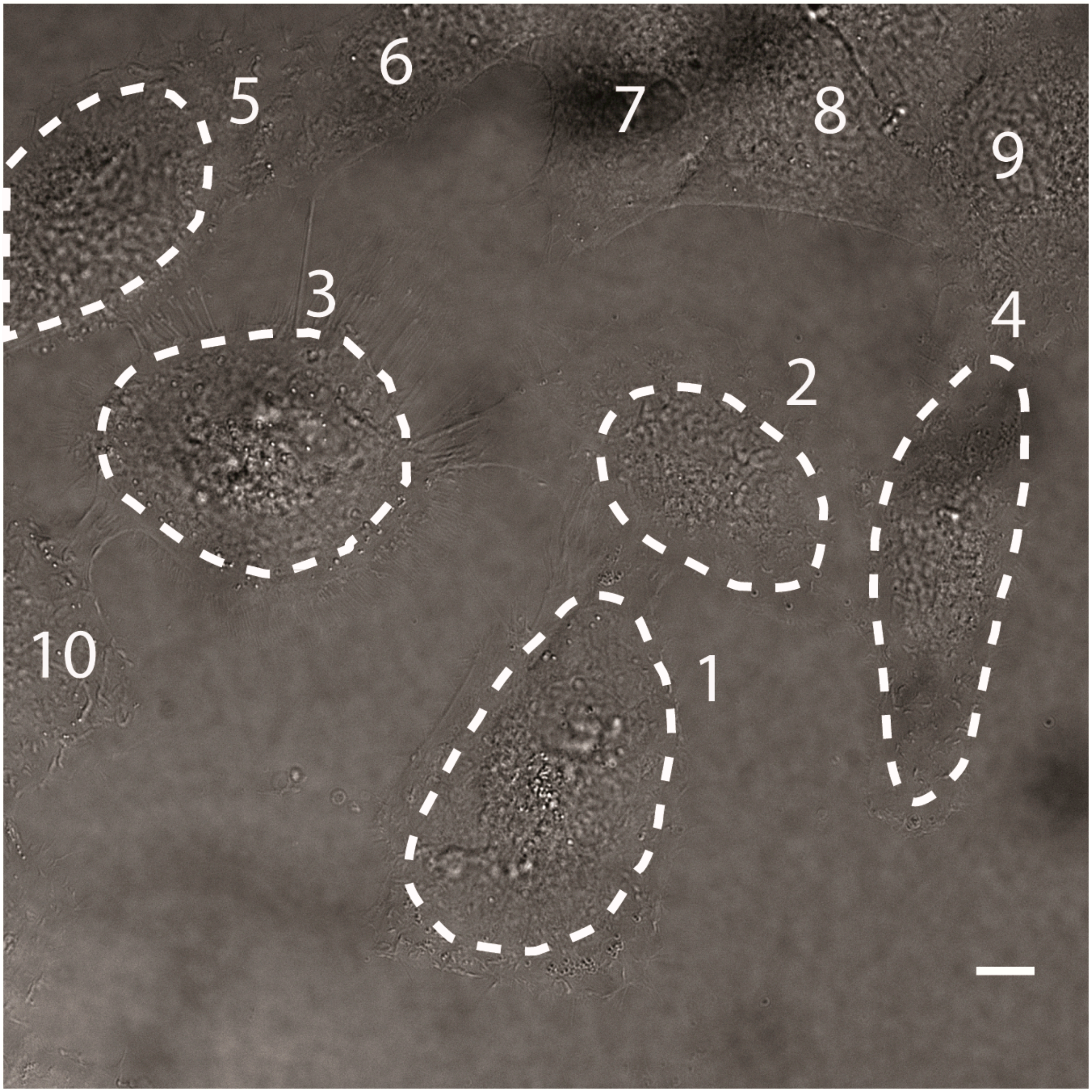

Figure S6 : Differential interference contrast (DIC) of HeLa cells stained with ***(E)*-3**/***(Z)*-3.** (Enlarged from figure 6A)

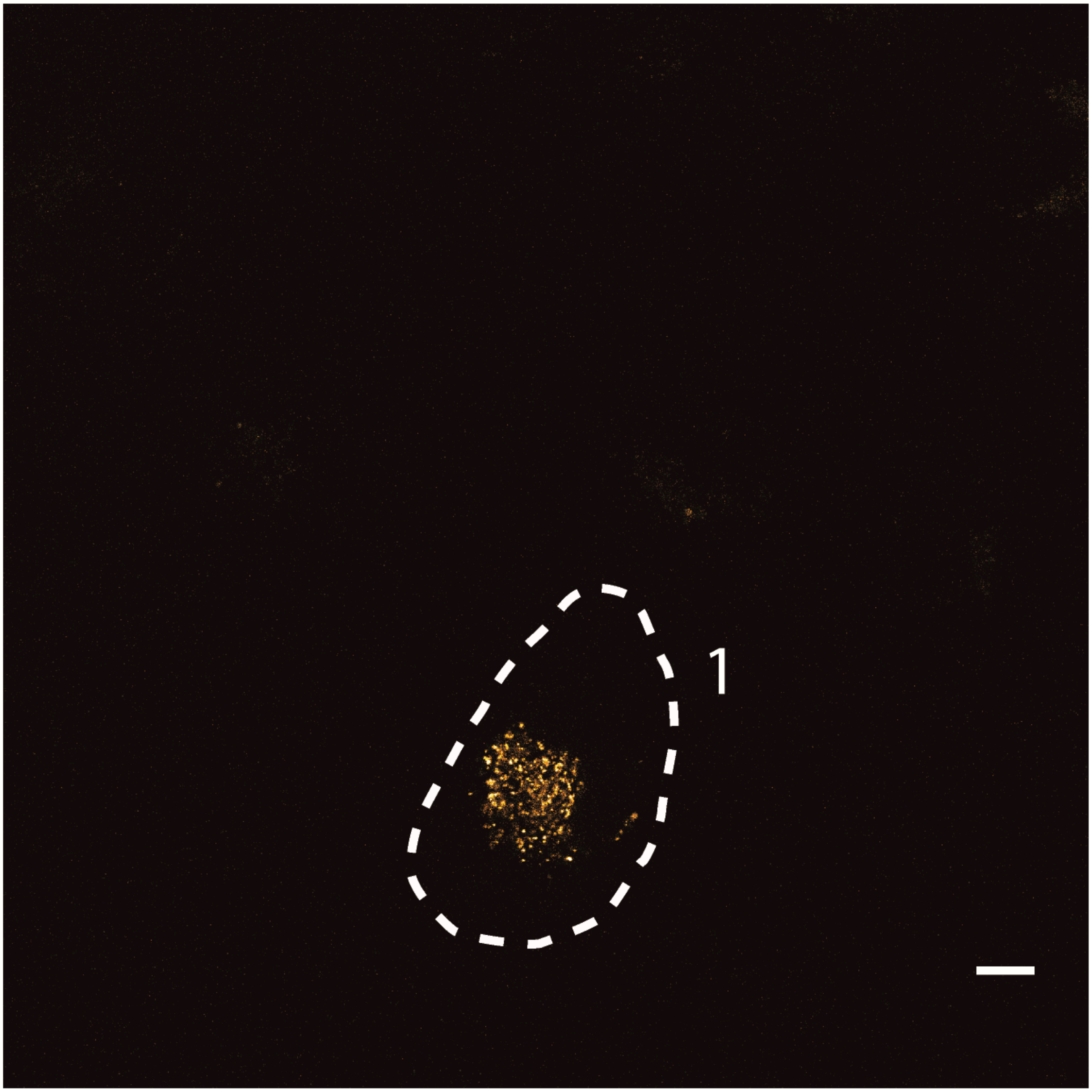

Figure S7 : Fluorescent image of HeLa cells stained with ***(E)*-3**/***(Z)*-3**. Cell 1 was selectively activated. (Enlarged from figure 6A)

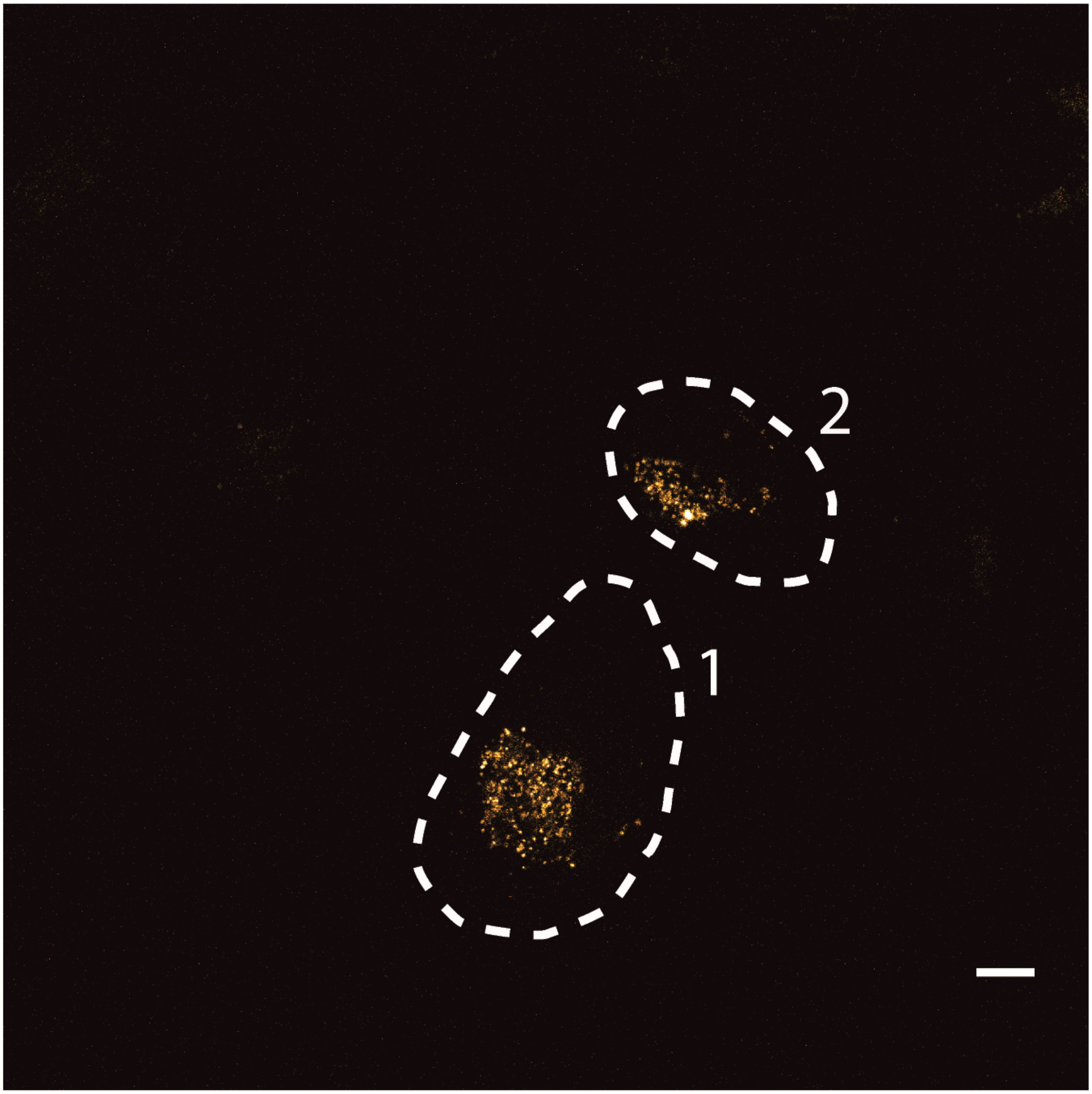

Figure S8 : Fluorescent image of HeLa cells stained with ***(E)*-3**/***(Z)*-3**. Cell 1 and 2 were selectively activated. (Enlarged from figure 6A)

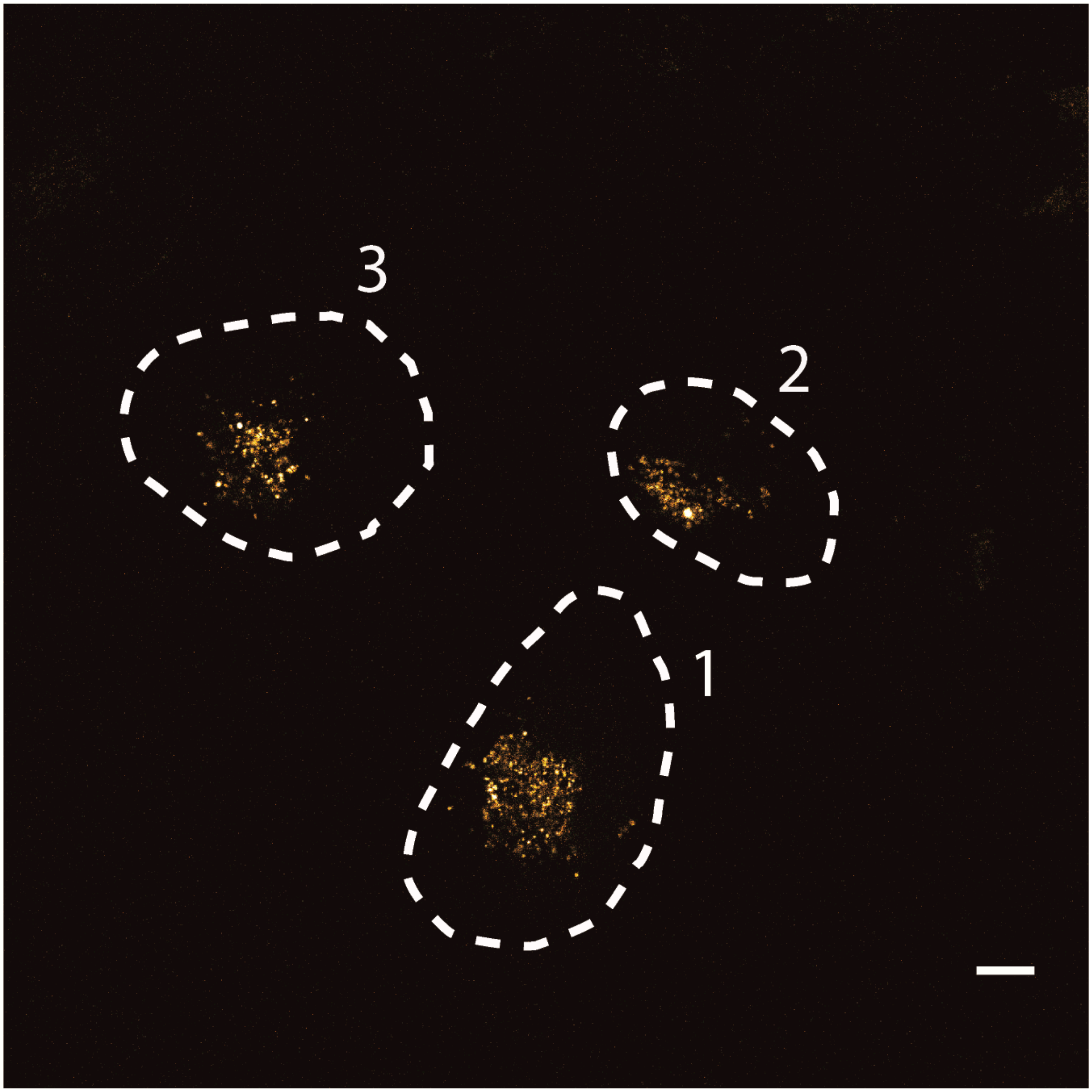

Figure S9 : Fluorescent image of HeLa cells stained with ***(E)*-3**/***(Z)*-3**. Cell 1, 2 and 3 were selectively activated. (Enlarged from figure 6A)

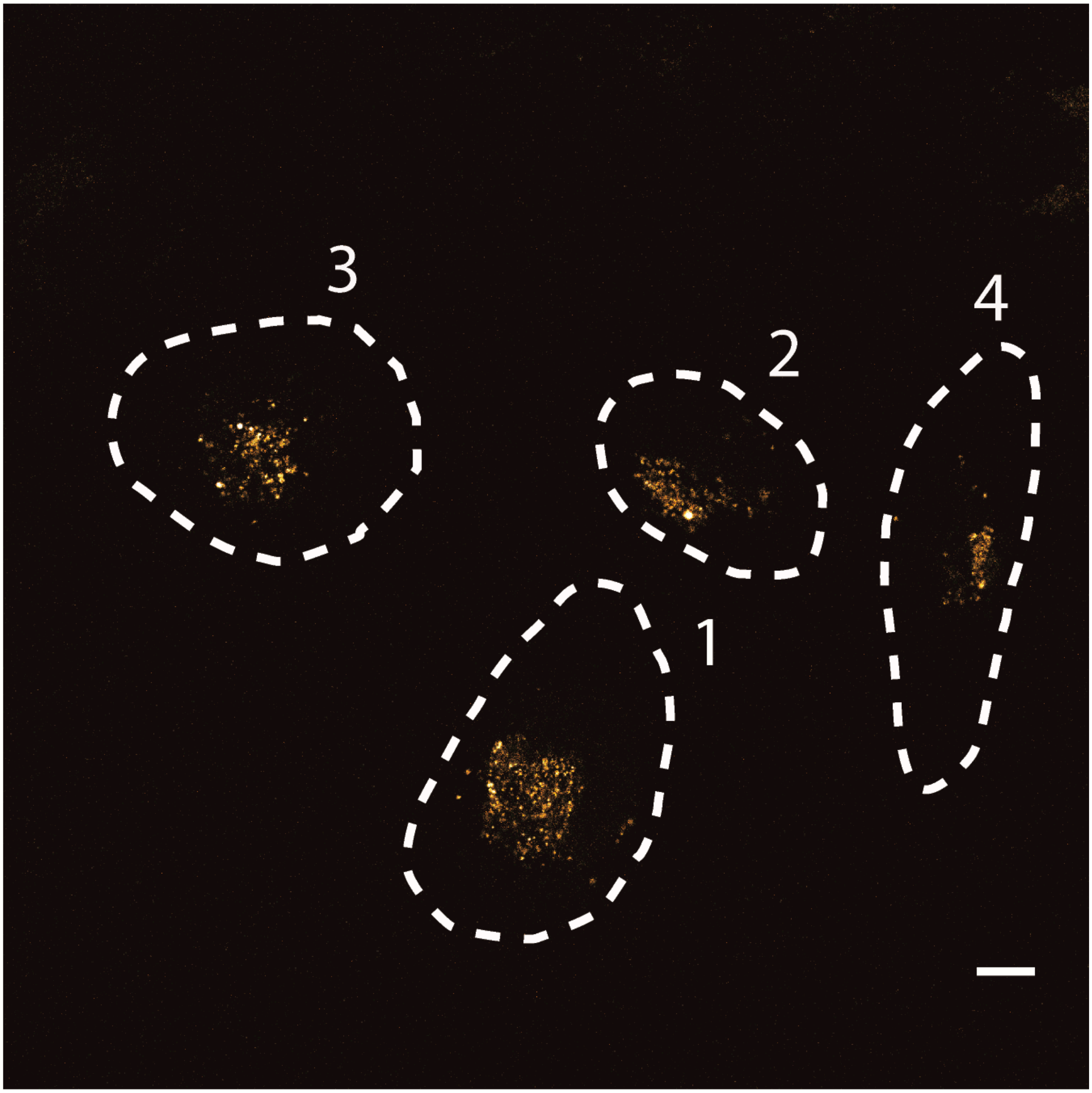

Figure S10 : Fluorescent image of HeLa cells stained with ***(E)*-3**/***(Z)*-3**. Cell 1, 2, 3, and 4 were selectively activated. (Enlarged from figure 6A)

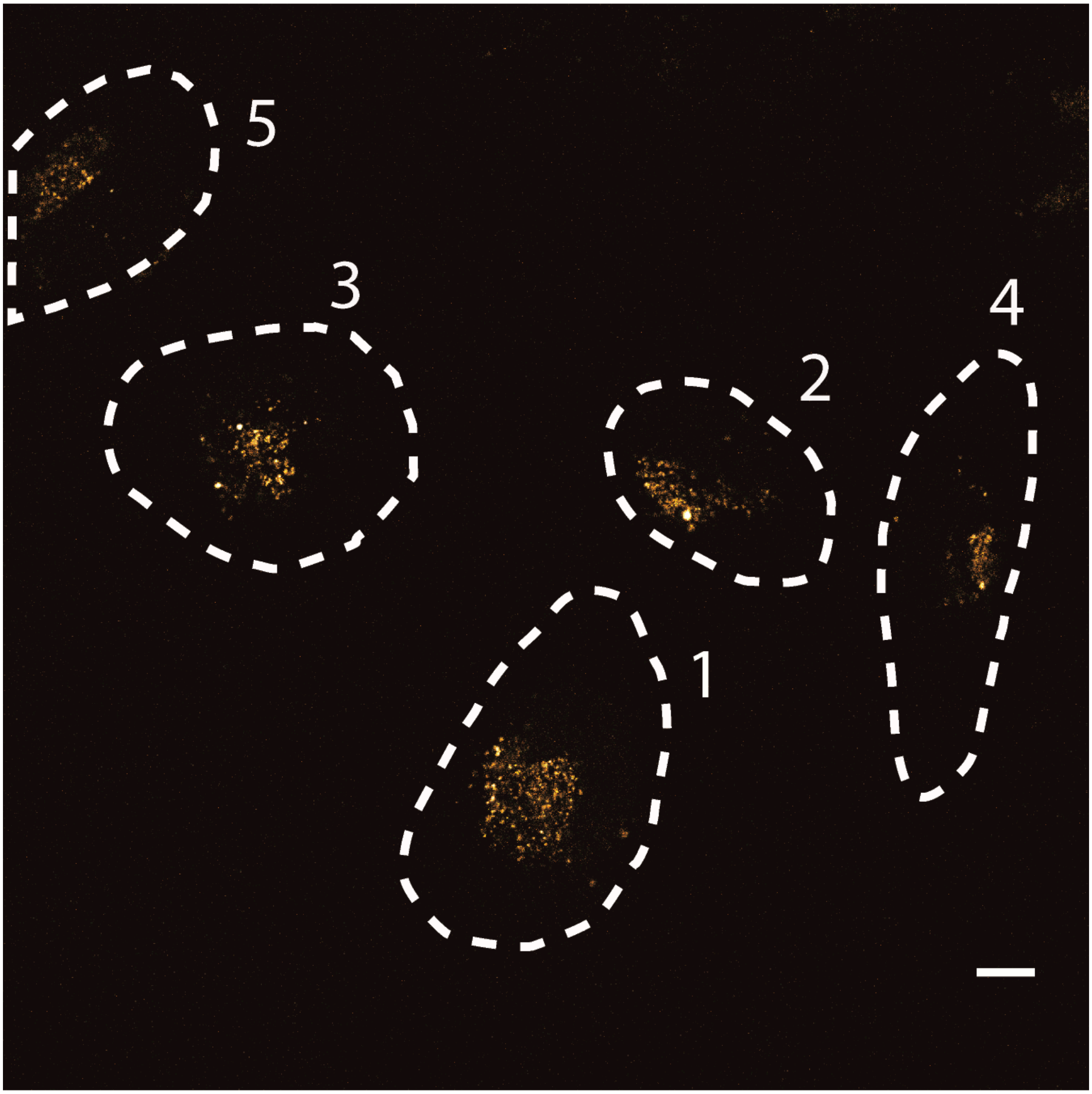

Figure S11 : Fluorescent image of HeLa cells stained with ***(E)*-3**/***(Z)*-3**. Cell 1, 2, 3, 4, and 5 were selectively activated. (Enlarged from figure 6A)

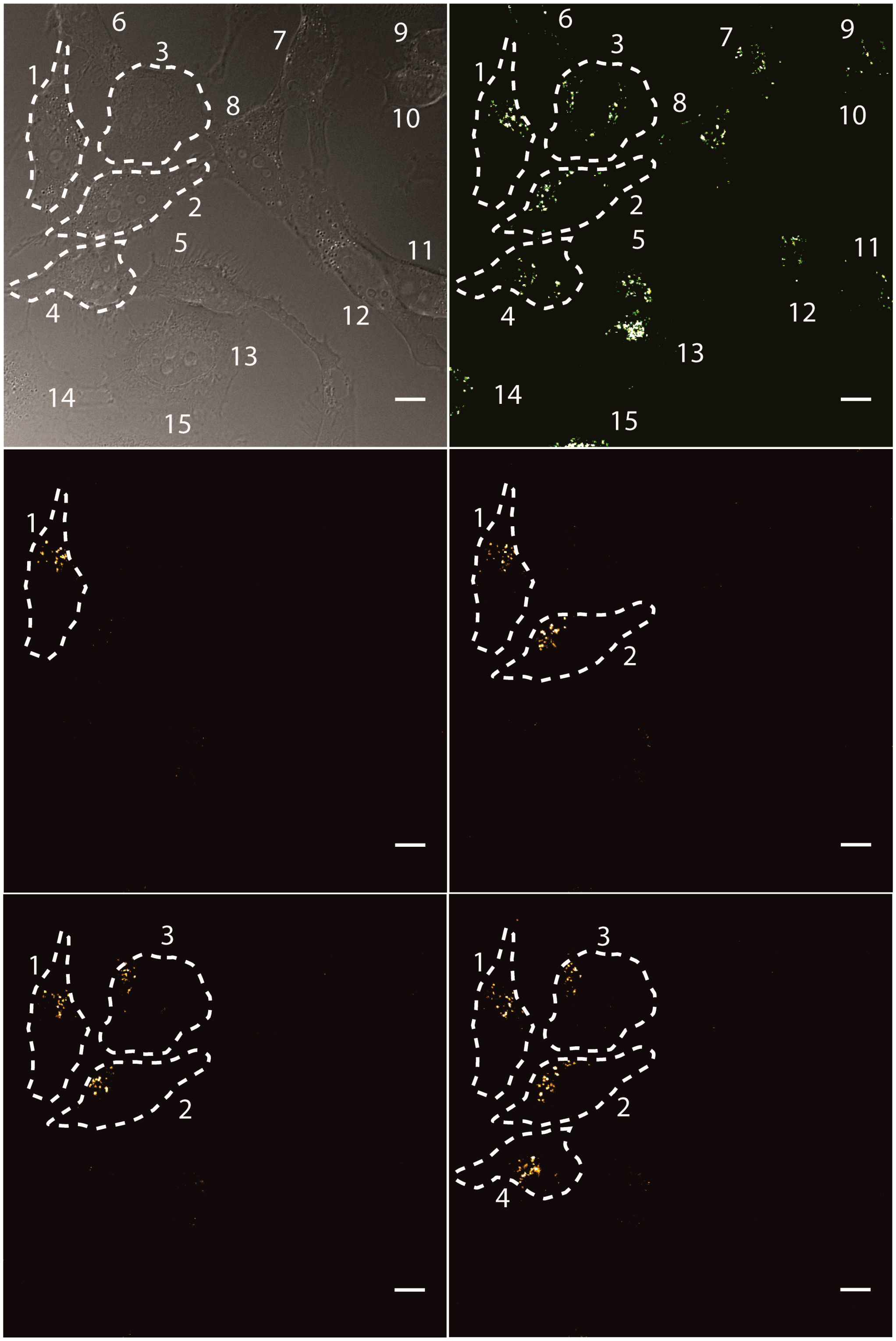

Figure S12 : Sequential activation of four individual cells (cell 1, 2, 3, and 4) in a field of fifteen HeLa cells. Dashed lines showed the cell periphery determined by DIC image. Each cell was activated by a 40-second irradiation using a 405 nm laser. Pre-activated signal was shown in green, observed by 405/525 channel. Post-activated signal was shown in orange, observed by 488/675 channel. Scale bar = 10 µm.

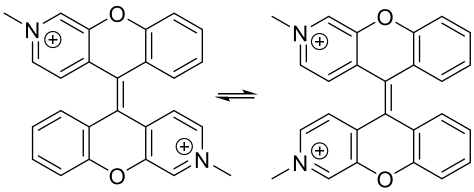

(*E*)-2 (*Z*)-2

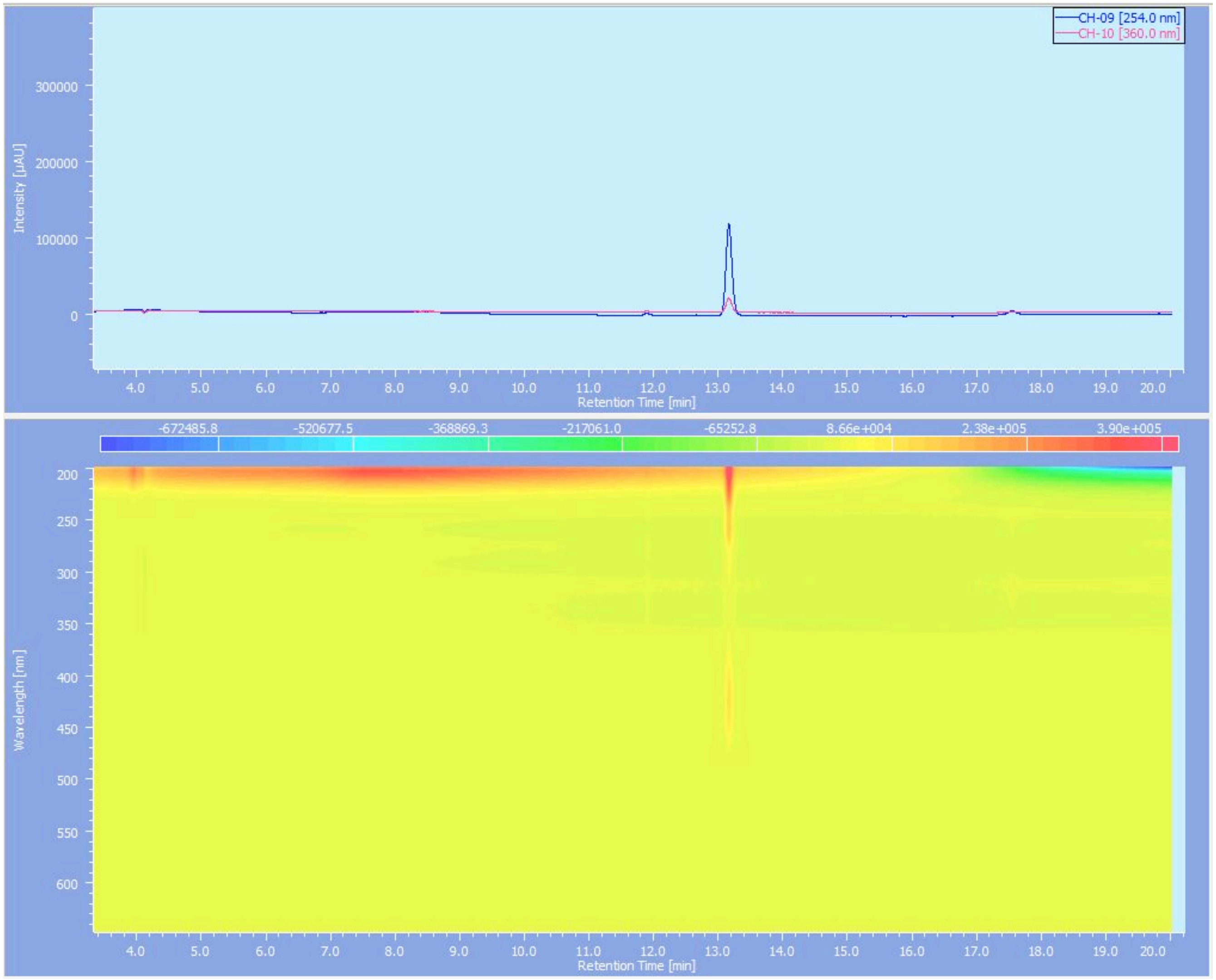

Figure S13. HPLC chromatogram of ***(E)*-2/*(Z)*-2**. Water containing 0.1% (v/v) trifluoroacetic acid and acetonitrile were used as eluents. Flow rate was 1 mL/min. Method time was 20 minutes.

Time (min)	% acetonitrile	Time (min)	% acetonitrile
0.00	0.0	15.00	100.0
1.00	0.0	17.80	100.0
13.00	50.0	18.00	0.0

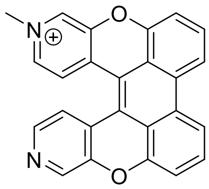

4

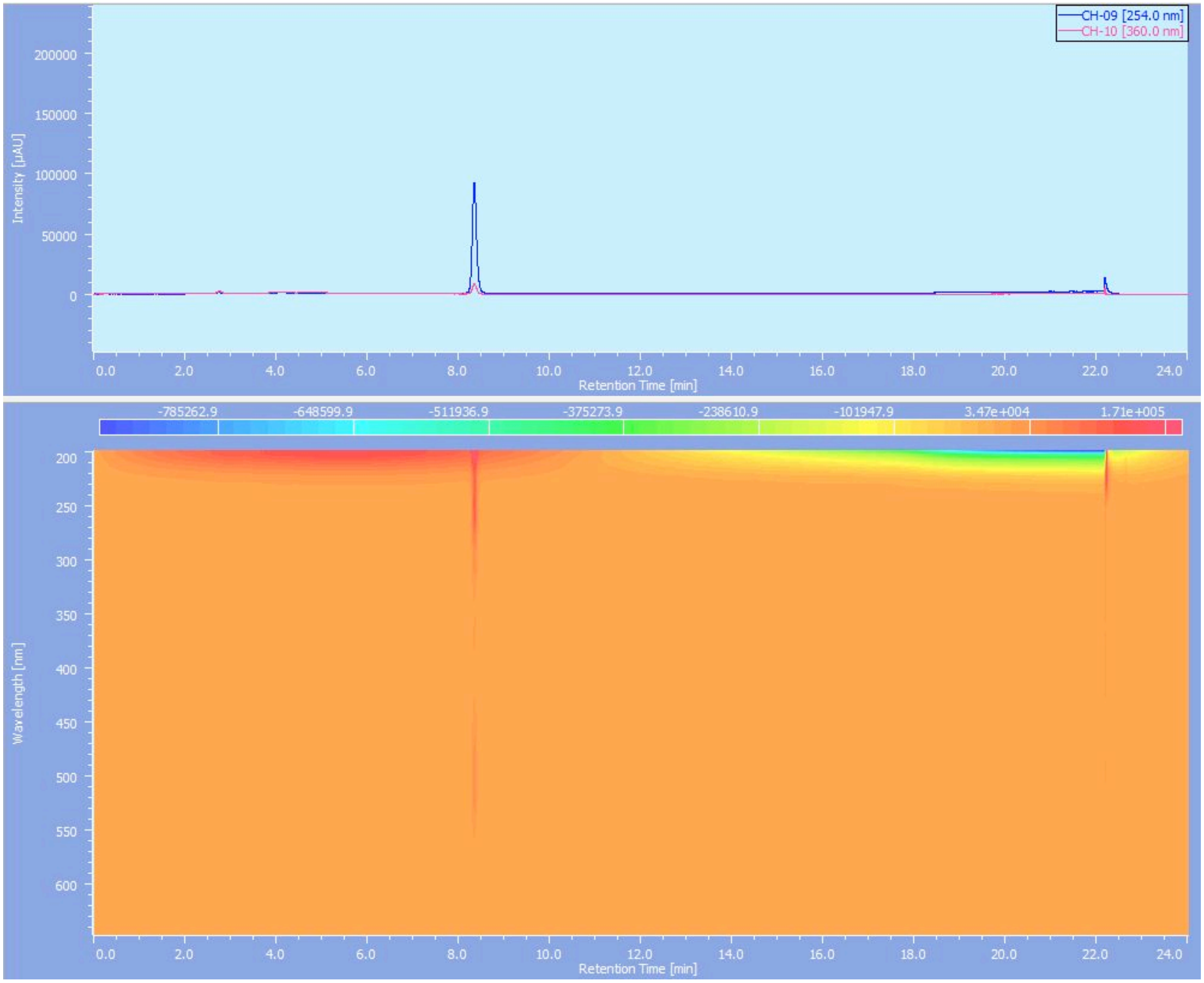

Figure S14. HPLC chromatogram of **4**. Water containing 0.1% (v/v) trifluoroacetic acid and acetonitrile were used as eluents. Flow rate was 1 mL/min. Method time was 24 minutes.

Time (min)	% acetonitrile	Time (min)	% acetonitrile
0.00	30.0	15.50	100.0
1.00	30.0	18.80	100.0
15.00	80.0	19.00	30.0

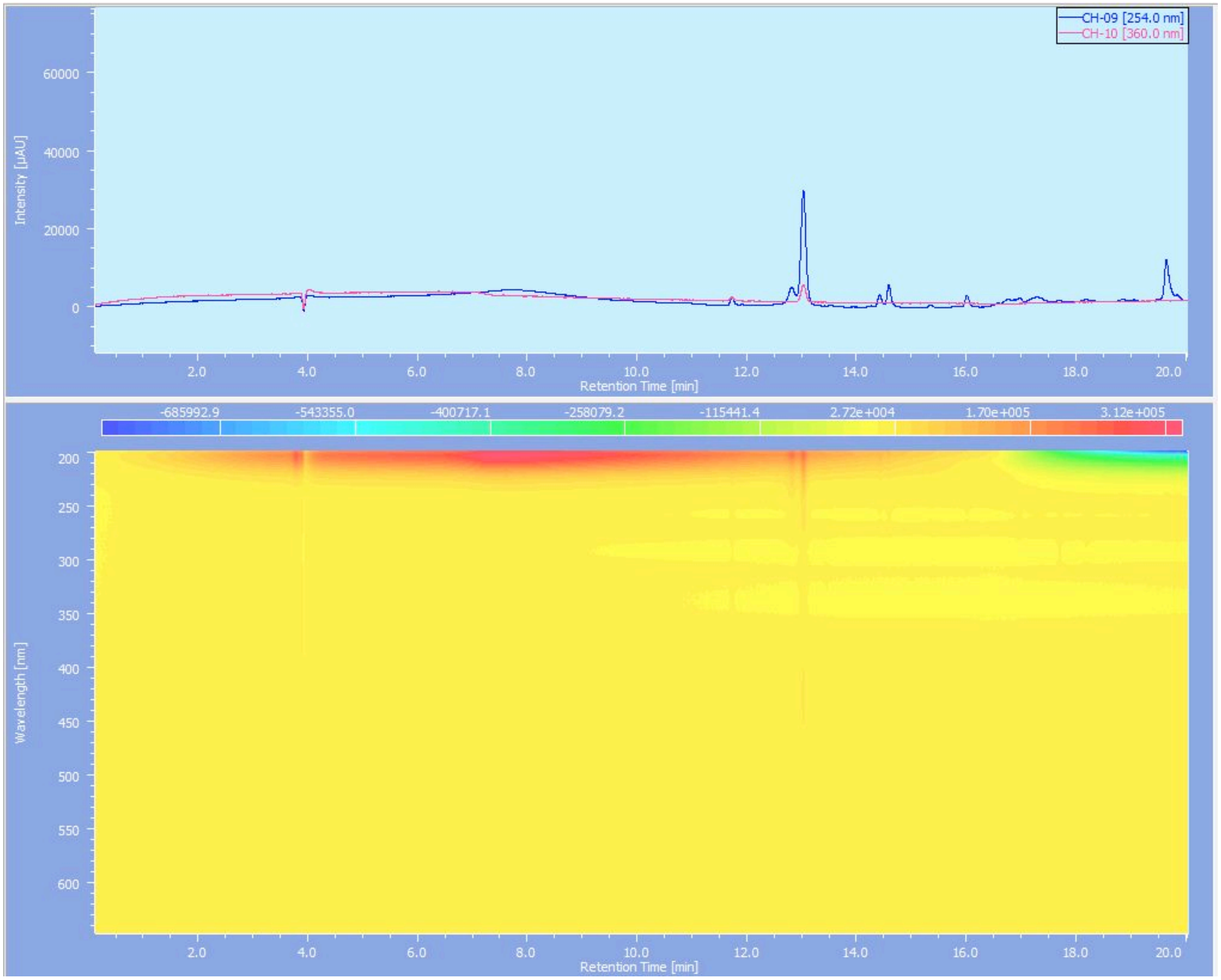

Figure S15. HPLC chromatogram of an aqueous solution of ***(E)*-2/*(Z)*-2** after 2 months at room temperature away from light. Water containing 0.1% (v/v) trifluoroacetic acid and acetonitrile were used as eluents. Flow rate was 1 mL/min. Method time was 20 minutes.

Time (min)	% acetonitrile	Time (min)	% acetonitrile
0.00	0.0	15.00	100.0
1.00	0.0	17.80	100.0
13.00	50.0	18.00	0.0

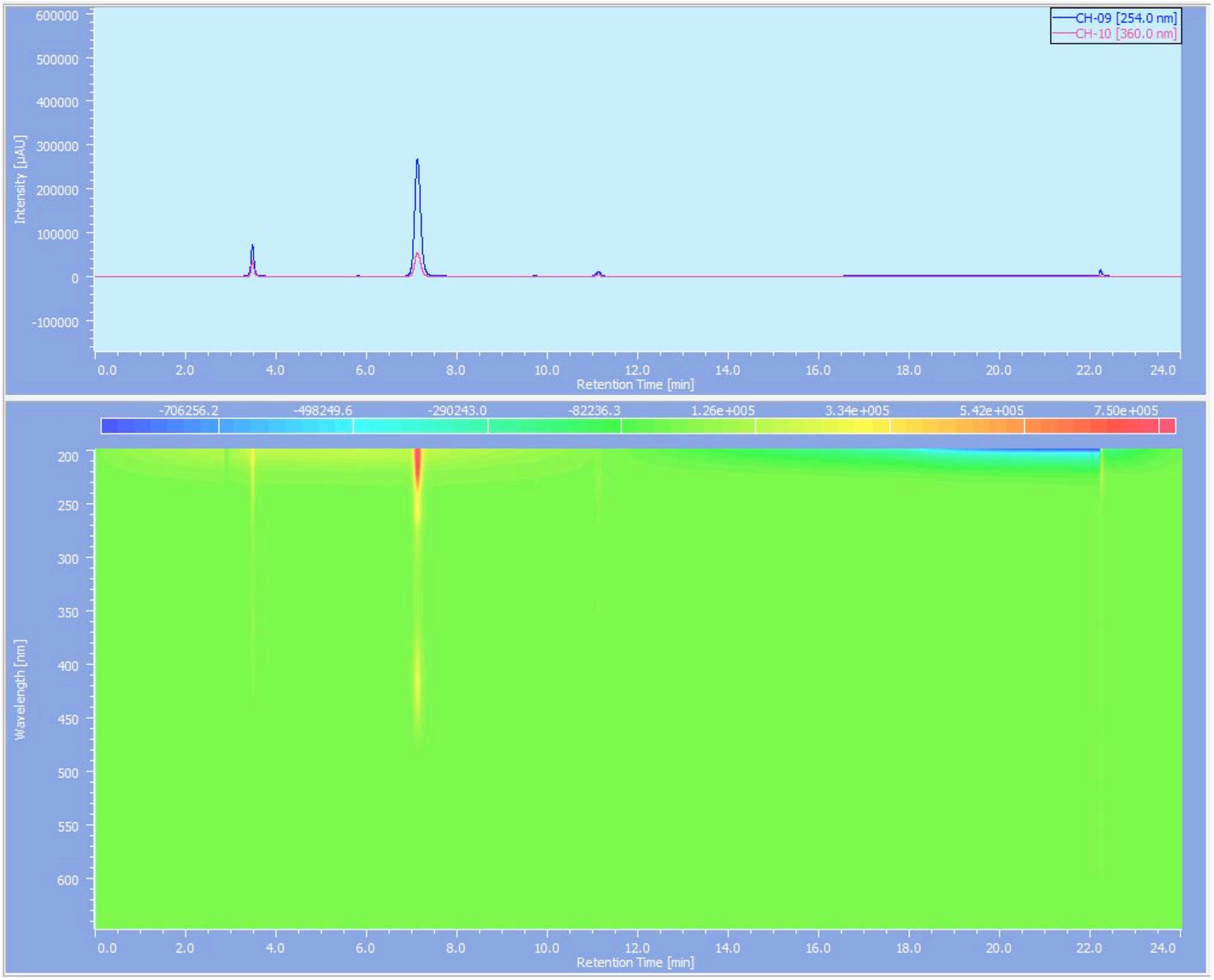

Figure S16. HPLC chromatogram of a 1 mM aqueous solution of ***(E)*-3/*(Z)*-3** after 2 months at room temperature away from light. Water containing 0.1% (v/v) trifluoroacetic acid and acetonitrile were used as eluents. Flow rate was 1 mL/min. Method time was 24 minutes.

Time (min)	% acetonitrile	Time (min)	% acetonitrile
0.00	30.0	15.50	100.0
1.00	30.0	18.80	100.0
15.00	80.0	19.00	30.0

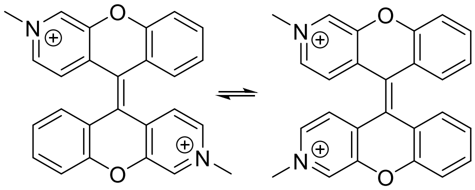

(*E*)-2 **(*Z*)-2**

Elemental Composition Report **Page 1**

Single Mass Analysis
Tolerance = 5.0 PPM / DBE: min = -1.5, max = 50.0
Element prediction: Off
Number of isotope peaks used for i-FIT = 3

Monoisotopic Mass, Odd and Even Electron Ions
50 formula(e) evaluated with 1 results within limits (up to 50 best isotopic matches for each mass)
Elements Used:
C: 0-50 H: 0-100 N: 0-2 O: 0-2
U_Penn_MaiT_DiMexyloR 87 (4.294)
1: TOF MS ES+

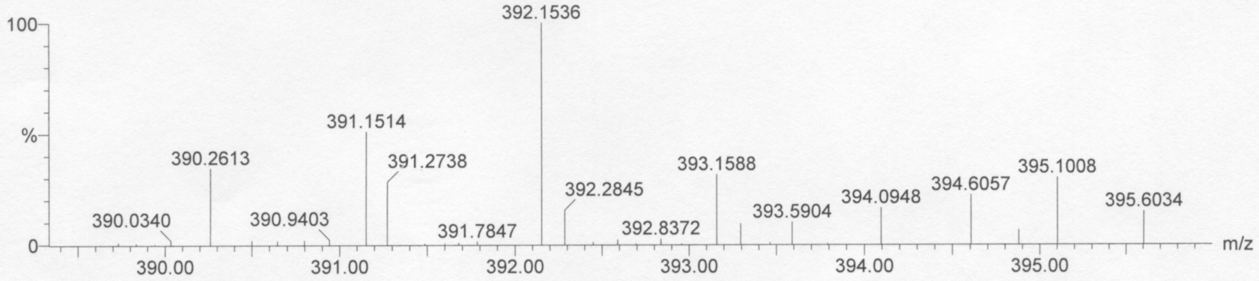

Minimum:				-1.5		
Maximum:		5.0	5.0	50.0		
Mass	Calc. Mass	mDa	PPM	DBE	i-FIT	Formula
392.1536	392.1525	1.1	2.8	18.0	n/a	C26 H20 N2 O2

Figure S17: Mass spectrum of ***(E)*-2/*(Z)*-2**.

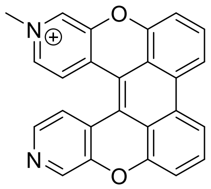

4

Elemental Composition Report

Page 1

Single Mass Analysis

Tolerance = 5.0 PPM / DBE: min = -2.0, max = 80.0
Element prediction: Off
Number of isotope peaks used for i-FIT = 2

Monoisotopic Mass, Even Electron Ions
174 formula(e) evaluated with 2 results within limits (all results (up to 1000) for each mass)
Elements Used:
C: 0-60 H: 0-100 N: 0-2 O: 0-5 Na: 0-1
12-Sep-2014
Penn_MaiT_4290_2 11 (0.973) Cm (11)

1: TOF MS ES+
3.50e+005

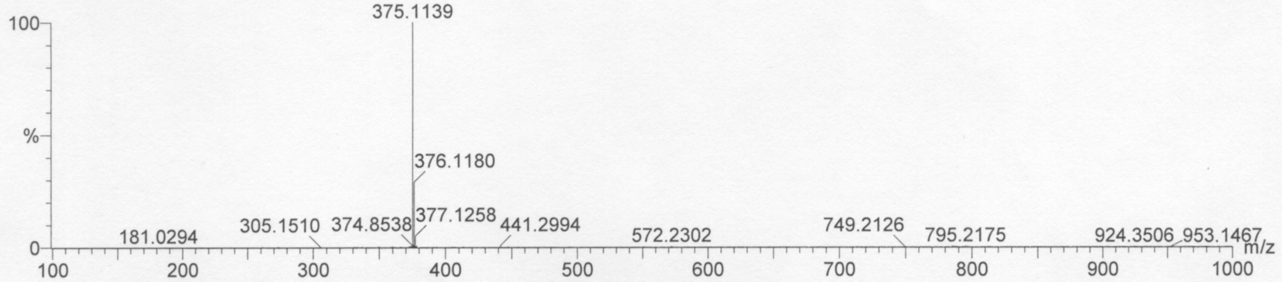

Minimum:				-2.0		
Maximum:		5.0	5.0	80.0		
Mass	Calc. Mass	mDa	PPM	DBE	i-FIT	Formula
375.1139	375.1134	0.5	1.3	19.5	6.2	C25 H15 N2 O2 ←
	375.1150	-1.1	-2.9	20.5	304.5	C28 H16 Na

Figure S18: Mass spectrum of **4**.

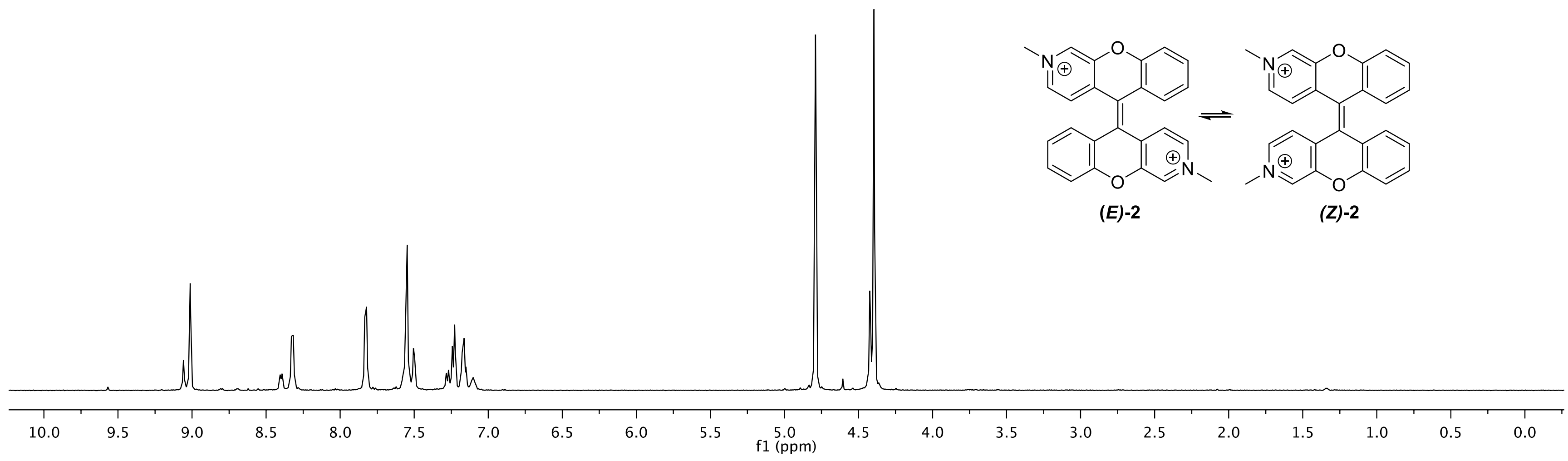
(E)-2
(Z)-2
f1 (ppm)

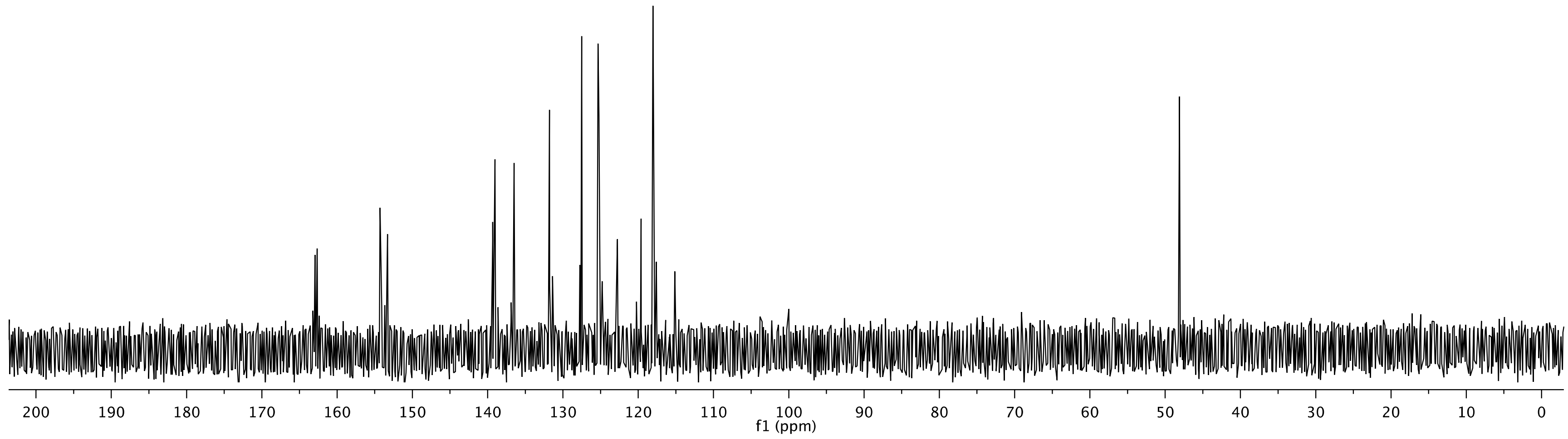
f1 (ppm)

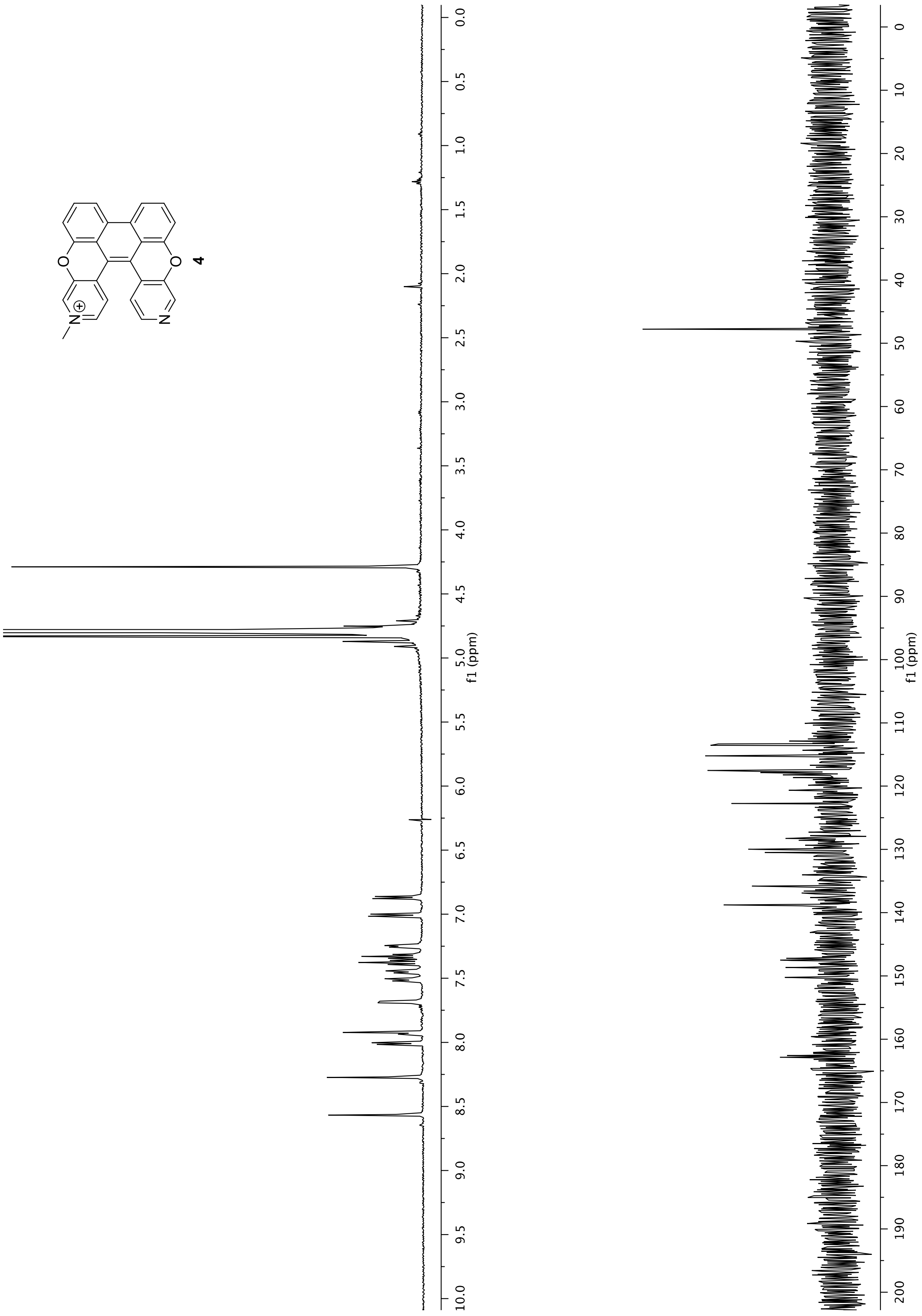
4
f1 (ppm)
f1 (ppm)
